# The Euchromatic and Heterochromatic Landscapes Are Shaped by Antagonizing Effects of Transcription on H2A.Z Deposition

**DOI:** 10.1371/journal.pgen.1000687

**Published:** 2009-10-16

**Authors:** Sara Hardy, Pierre-Étienne Jacques, Nicolas Gévry, Audrey Forest, Marie-Ève Fortin, Liette Laflamme, Luc Gaudreau, François Robert

**Affiliations:** 1 Institut de recherches cliniques de Montréal, Montréal, Québec, Canada; 2 Département de biologie, Faculté des Sciences, Université de Sherbrooke, Sherbrooke, Québec, Canada; 3 Département de médecine, Faculté de Médecine, Université de Montréal, Montréal, Québec, Canada; The University of North Carolina at Chapel Hill, United States of America

## Abstract

A role for variant histone H2A.Z in gene expression is now well established but little is known about the mechanisms by which it operates. Using a combination of ChIP–chip, knockdown and expression profiling experiments, we show that upon gene induction, human H2A.Z associates with gene promoters and helps in recruiting the transcriptional machinery. Surprisingly, we also found that H2A.Z is randomly incorporated in the genome at low levels and that active transcription antagonizes this incorporation in transcribed regions. After cessation of transcription, random H2A.Z quickly reappears on genes, demonstrating that this incorporation utilizes an active mechanism. Within facultative heterochromatin, we observe a hyper accumulation of the variant histone, which might be due to the lack of transcription in these regions. These results show how chromatin structure and transcription can antagonize each other, therefore shaping chromatin and controlling gene expression.

## Author Summary

DNA in living cells is packaged into chromatin by histones and non-histone proteins. This packaging is very dynamic, allowing the controlled access of regulatory proteins such as transcription factors to DNA. Most chromatin is packaged with so-called canonical histones; namely H2A, H2B, H3, and H4. In some regions, however, variant histones replace canonical histones, creating special chromatin regions. Here we show that the variant histone H2A.Z is dynamically recruited to promoter regions where it helps in the recruitment of RNA polymerase II, the enzyme responsible for the first step of gene expression. In addition, we show that H2A.Z also associates randomly in the genome, but these molecules are removed during the passage of RNA polymerase II. In non-transcribed regions, H2A.Z accumulates in large domains called heterochromatin. We propose that a battle between random H2A.Z deposition and RNAPII-dependent H2A.Z eviction shapes the chromatin landscape.

## Introduction

Chromatin dynamics is now well recognized as key in the regulation of nuclear processes such as gene expression, DNA replication and DNA repair, therefore impinging on biological phenomena such as cellular differentiation as well as normal and cancer development. Cells have developed strategies to locally reconfigure chromatin structure, including i) the use of ATP-dependent chromatin remodeling complexes to either modify the topology, slide or remove nucleosomes; ii) the covalent modification of histone and; iii) the incorporation of variant histones within nucleosomes.

Incorporation of variant histones within chromatin regions is emerging as a way for cells to create specialized chromatin domains in order to mediate various cellular functions [1]. A large number of variant histones exist, especially for histone H2A and H3, but the best characterized variant histone is perhaps H2A.Z [2],[3]. H2A.Z is highly similar to canonical H2A. The two molecules diverge only by a few amino acids and the structure of a nucleosome containing H2A.Z is almost identical to that of a canonical nucleosome [4]. Despite this, H2A.Z clearly has a specialized function that cannot be complemented by an additional allele of H2A. H2A.Z is required for viability in most organisms [5]–[8]. It is necessary for early development in *Xenopus*, *Drosophila* and mouse [6]–[8], is associated with cancer progression [9]–[12], and is required for estrogen receptor signaling [13] and for embryonic stem cell lineage commitment [14].

H2A.Z occupies one or two nucleosomes in most promoters, a phenomenon first described in yeast [15]–[19] but recently shown to be conserved in *Drosophila*, chicken, plants and mammals [20]–[25]. In mammalian cells, H2A.Z also occupies pericentromeric heterochromatin domains [26] and regulatory elements such as enhancers and insulators [20],[22]. The involvement of H2A.Z in gene regulation has been clearly established [24], [27]–[30] but the mechanism underlying its function is not fully understood. Work in yeast suggest that H2A.Z regulates transcription either by creating unstable nucleosomes [19], by positioning nucleosomes [15], by making contacts with the transcription machinery [27] and/or by maintaining active genes to the nuclear periphery [31]. In mammalian cells, H2A.Z was shown to be important for transactivation by transcription factors such as p53 [24] and nuclear receptors [13],[32], leading to local chromatin reorganization and transcriptional regulation. A possible role for H2A.Z in transcriptional elongation has also been reported [30]. Despite these few documented cases, it is not known whether H2A.Z plays a more general role in transcription.

Here we provide evidence for a global role of H2A.Z in gene expression in human cells. We also demonstrate that H2A.Z regulates early steps in transcription by helping in the recruitment or RNA polymerase II (RNAPII) to promoters. Surprisingly, we also show that a mechanism allows for the non-targeted incorporation of H2A.Z molecules in the genome, and that transcription results in the depletion of these randomly incorporated H2A.Z molecules from genes. The battle between these two phenomena shapes the chromatin landscape and may have implications in heterochromatin function.

## Results

### Human H2A.Z associates with promoter region

**Figure 1:**
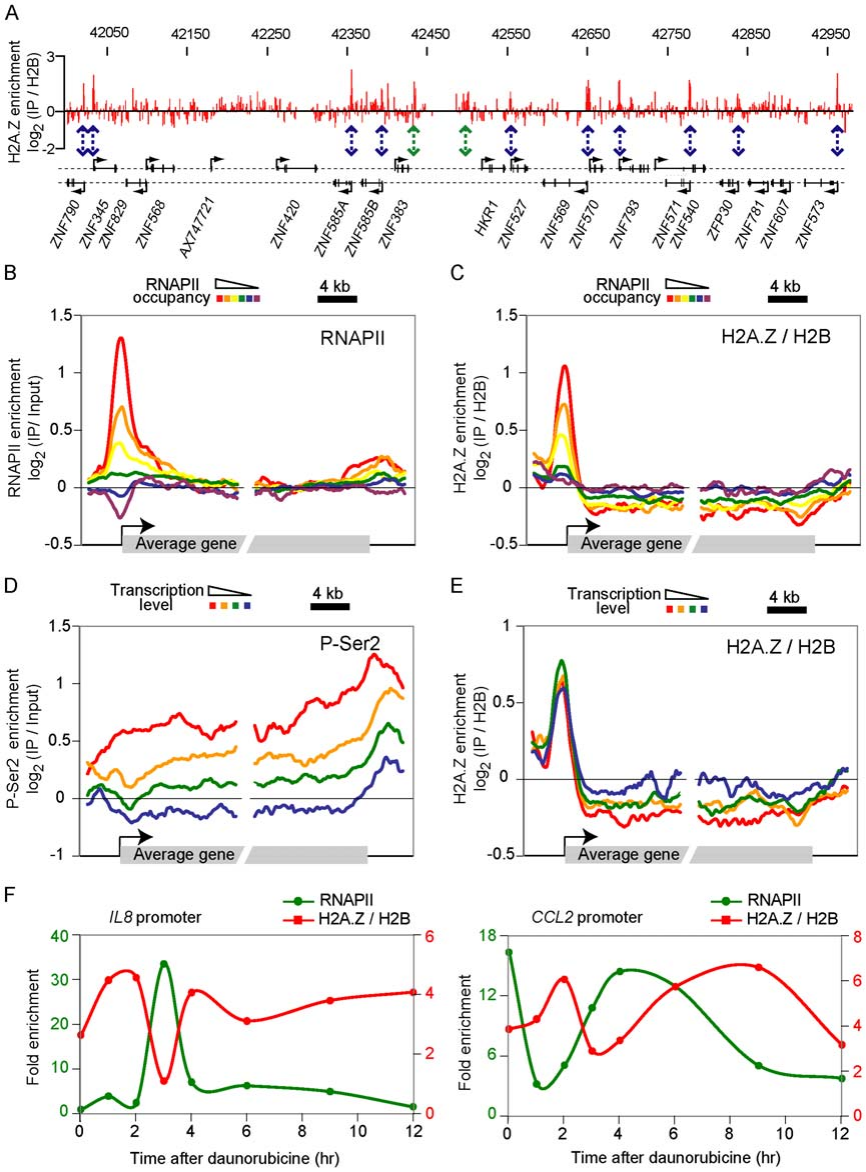
Human H2A.Z associates with promoters early during the transcription process. (A) A snapshot showing H2A.Z enrichment (red) along a 1 Mb region from chromosome 19. Chromosomal coordinates (in kb) are shown on top and genes are shown at the bottom. Double-headed dashed arrows are pointing to H2A.Z peaks that map to gene promoters (blue) and other elements (green). (B) Mapping of RNAPII enrichment on groups of genes defined by their occupancy of RNAPII at their promoter (Figure S3A). (C) Mapping of H2A.Z on the same groups of genes as panel B. (D) Mapping of P-Ser2 RNAPII enrichment on groups of genes defined as follow: genes with high RNAPII occupancy at their promoter (the union of the red, orange and yellow genes from panel B were ranked based on their P-Ser2 RNAPII level and then separated into four groups. The red genes correspond to genes with high level of RNAPII and high level of P-Ser2 RNAPII. The blue genes have high level of RNAPII but low level of P-Ser2 RNAPII. (E) H2A.Z mapping on the gene groups defined in panel D. (F) RNAPII and H2A.Z enrichment over time after dauno treatment in serum-deprived (G1/G0-arrested) cells on the promoter of *IL8* (left) and *CCL2* (right). Note that a version of that figure where all ZNF genes were removed is shown as Figure S12).

In order to gain some understanding about the function of H2A.Z in human cells, we performed ChIP-chip experiments in U2OS cells. The H2A.Z-enriched material was hybridized on tiling arrays covering the non-repetitive portion of chromosome 19 and 22 at an average of 250 base pairs resolution. Acetylated-H2A.Z, RNAPII, P-Ser2 RNAPII as well as various histone modifications were also profiled in the same conditions (summarized in Table S1 and available in Dataset S1, S2, S3, S4, S5). H2A.Z occupancy is high in the 5′ regions of many genes (Figure 1A and Figure S1), reminiscent of what is observed in yeast [15]–[19] and in agreement with work in human, plants and *Drosophila* by others [20],[21],[23]. H2A.Z is also enriched in distal regulatory elements (as defined as regions enriched in H3K4me1 [33]) (Figure S2) as described before [20], and in heterochromatin (see below) as previously suggested [34]. Interestingly, when genes were binned based on their RNAPII occupancy (Figure 1B and Figure S3A), a clear correlation was observed between the level of RNAPII and that of H2A.Z at promoters (Figure 1C and Figure S3B). This is different from what we and others have observed in yeast, where H2A.Z is present at most promoters regardless of their transcriptional activity [15],[16],[19], but agrees with previous reports from metazoan systems [20],[21]. Taken together, our data shows that H2A.Z associates with many kinds of regulatory elements in human cells, and that its association with promoters is linked to RNAPII.

### H2A.Z is dynamically recruited to promoters prior to RNAPII

It has recently been observed that a large fraction of metazoan genes are associated with RNAPII molecules paused at their 5′ end [35],[36]. Consequently, the level of RNAPII at promoters does not strictly correlate with transcription in mammalian cells. We used two independent strategies to separate active genes from genes poised by the presence of paused RNAPII. First, we binned the genes with high levels of RNAPII at promoters into four groups based on their level of histone H3K36me3 (Figure S4). Levels of H3K36me3 have been previously shown to represent a good proxy for transcription rate [37]. Genes with high levels of both RNAPII and H3K36me3 are considered as actively transcribed [37] since H3K36 is methylated co-transcriptionally. On the other hand, genes with high levels of RNAPII but low levels of H3K36me3 are generally considered as non-transcribed [37], and most likely represent genes with paused RNAPII, a process that may poise genes for rapid activation [35],[36]. Second, we profiles RNAPII phosphorylated on Ser2. Because phosphorylation of Ser2 is occurring during elongation [38], the level of P-Ser2 RNAPII on genes should be a better indication of transcription rate than that of total RNAPII at promoters. As expected P-Ser2 RNAPII correlates better with levels of H3K36me3 (Pearson, r = 0,53) than with levels of RNAPII (Pearson, r = 0.34). Interestingly, H2A.Z is present at similar levels on transcribed genes and on genes poised for activation by the presence of paused RNAPII (Figure 1E and Figure S5, using P-Ser2 and Figure S4, using H3K36me3). This data demonstrates that H2A.Z incorporation at promoters does not require fully processive transcription. Our data rather suggests that H2A.Z is recruited early during the transcription cycle; that is, just prior to, or concomitantly with, RNAPII recruitment.

Expression profiling experiments identified *IL8* and *CCL2* as two genes transiently induced upon daunorubicin (dauno) treatment, a DNA damaging agent known to induce p21 expression via the p53 pathway [39] (Figure S6). To investigate the dynamics of the interaction of H2A.Z with promoters, we looked at the association of the variant histone with the promoters of *IL8* and *CCL2* upon treatment of U2OS cells with dauno. A detailed time course looking at H2A.Z (normalized for H2B) and RNAPII recruitment at *IL8* and *CCL2* showed that H2A.Z is recruited to the promoter just prior to RNAPII and leaves the promoter as the polymerase gets recruited (Figure 1F and Figure S7 for additional replicates). Interestingly, at *CCL2*, RNAPII is present in a paused state at the promoter prior to dauno treatment. Upon activation, RNAPII is first released from the promoter; an event that is coupled to an increase in H2A.Z occupancy and followed by re-loading of RNAPII. Note that these variations in H2A.Z levels at promoters cannot be explained simply by fluctuations in nucleosome density since our data are normalized for nucleosome occupancy. Taken together our data suggests that H2A.Z associates with promoters prior to RNAPII and may therefore affect early steps in transcription such as RNAPII recruitment.

### H2A.Z potentiates the recruitment of RNAPII to promoters

**Figure 2:**
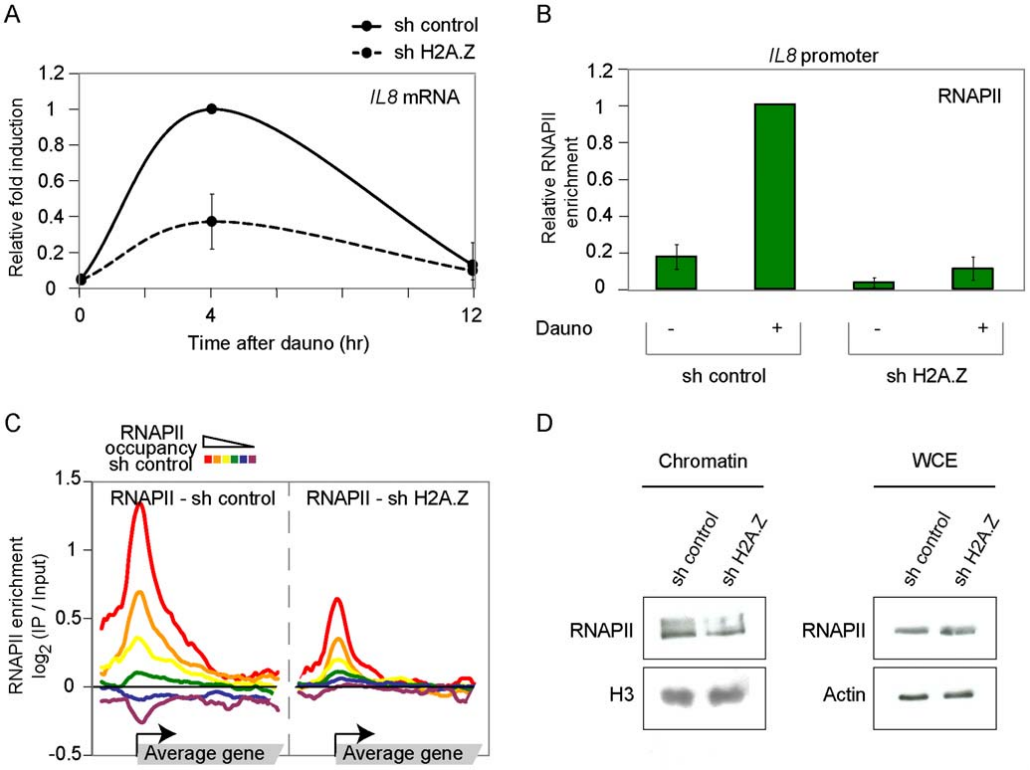
Human H2A.Z assists in the recruitment of RNAPII to promoters. (A) Expression of *IL8* upon treatment with dauno. Prior to addition of dauno, cells were transfected with either H2A.Z-specific shRNA (dashed line) or control shRNA (solid line) expressing plasmids. The experiment was repeated twice and replicates were combined. The data are expressed as the fraction the maximum induction. (B) RNAPII occupancy at the *IL8* promoter after dauno treatment. Cells where treated as in panel A and the association of RNAPII with the *IL8* promoter was monitored by ChIP followed by QPCR at 0 and 4 hrs of treatment with dauno. The experiment was repeated twice and replicates were combined. The data are expressed as the fraction the maximum enrichment. (C) Mapping of the RNAPII on groups of genes defined by their RNAPII occupancy levels on cells transfected with control shRNA. The RNAPII enrichment is shown for H2A.Z-specific shRNA (left) and control shRNA (right) expressing plasmids. (D) Knockdown of H2A.Z causes a global decrease of RNAPII associated with bulk chromatin. Chromatin extracts and Whole cell extracts (WCE) were prepared from U2OS cells treated with control shRNAs or H2A.Z-specific shRNAs. Extracts were separated on SDS-PAGE gel, transfer on membrane and probed for RNAPII (8WG16), H2A.Z, actin and H2B.

In order to test for a role of H2A.Z in RNAPII recruitment, we made use of small hairpin-mediated RNA (shRNA) interference. Knockdown of H2A.Z crippled both the induction of *IL8* (Figure 2A and Figure S8A) and the recruitment of RNAPII (Figure2B and Figure S8B) upon induction by dauno. This shows that H2A.Z plays a role in the regulation of *IL8* by dauno by helping with the recruitment of RNAPII. This mechanism is consistent with what is observed in yeast where we showed that H2A.Z (Htz1 in yeast) assists in the recruitment of RNAPII and its associated co-factors [27],[29],[40]. Interestingly, knockdown of H2A.Z also resulted in reduction of RNAPII occupancy at the genomic level in steady state cells (Figure 2C and Figure S9), arguing for a broad role for H2A.Z in RNAPII recruitment in metazoans. This data is supported by the fact that the amount of RNAPII associated with bulk chromatin is reduced in H2A.Z knockdown cells, despite the fact that no change in total cellular RNAPII levels are observed (Figure 2D). To our knowledge, this is the first report of such a broad effect of H2A.Z in transcriptional regulation, although a transcriptional role has been demonstrated for a few genes [24],[32].

### H2A.Z is actively depleted from transcribed genes

**Figure 3:**
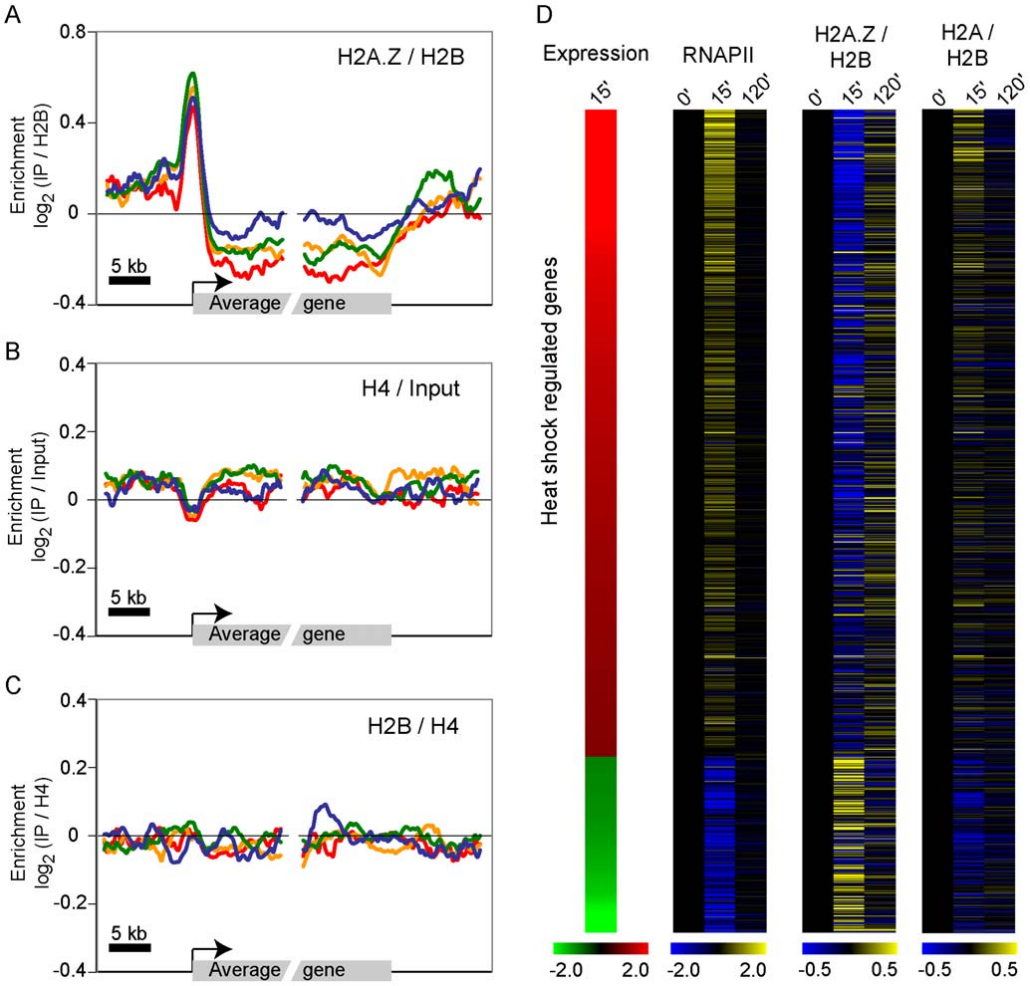
Transcription results in a depletion of H2A.Z from transcribed regions. (A) Zoomed out view of Figure 1E. (B) H4 enrichment (normalized to total genomic DNA) on the gene groups defined in Figure 1D. (C) H2B enrichment (normalized to H4) on the gene groups defined in Figure 1D. (D) Yeast cells were subjected to a heat shock and the occupancy of RNAPII, H2A.Z, H2A, and H2B was monitored across the whole genome over time by ChIP-chip. The RNAPII/Input ,H2A.Z/H2B and H2A/H2B occupancy levels (relative to time 0) is shown on gene body of all genes induced or repressed by heat shock treatment according to previously published data [56]. The expression fold change is also shown on the left. All yeast ChIP-chip data are available in Dataset S6.

Close inspection of Figure 1E and Figure S4D revealed that H2A.Z is depleted from the body of actively transcribed genes compared to inactive genes. Figure 3A shows a zoomed out view of Figure 1E allowing to fully appreciating the observation that transcribed genes have lower H2A.Z levels than their flanking intergenic regions (the regions upstream of the promoter and downstream of the terminator). This data shows that H2A.Z accumulates randomly in the genome at low levels and suggests that transcription may “clean” genes from these “miss-incorporated” histone variant molecules. Because our H2A.Z profiles are normalized for H2B occupancy, it seems unlikely that this result is solely due to nucleosome loss during transcription but we nevertheless investigated that possibility in more details. First, we looked at nucleosome occupancy by profiling histone H4 normalized for total genomic DNA. Histone H4 occupancy is not affected by transcription in these conditions (Figure 3B), ruling out the possibility that transcription results in a depletion of H3/H4 tetramer. H2B occupancy, when normalized for histone H4 levels (Figure 3C) or for total genomic DNA (not shown), is not dramatically affected by transcription either, suggesting that H2A/H2B dimers are not depleted by transcription. Since it is well known that transcription disrupts and reassembles H2A/H2B dimers [41], we conclude that H2A.Z depletion likely results from a preferable re-association of H2A-containing dimers over H2A.Z-containing dimers in the wake of RNAPII (see Discussion). Interestingly, revisiting our previously published ChIP-chip data on yeast H2A.Z suggested that this phenomenon is conserved throughout eukaryotes (Figure S10). We therefore took advantage of the tractable yeast system to investigate H2A.Z eviction in a dynamic fashion. Yeast cells were submitted to a heat shock and H2A.Z occupancy was measured by ChIP-chip over time. H2A was also profiled as a control. Heat shock was chosen because it strongly induces a significant number of genes in a transient manner, allowing us to look at both activation (between 0 and 15 minutes) and repression (between 15 minutes and 2 hours). As shown in Figure 3D, H2A.Z levels (normalized to H2B) are reduced in the transcribed regions of heat shock-induced genes (compare 0 minutes with 15 minutes). This data clearly shows that transcription directly or indirectly depletes H2A.Z molecules during elongation. In addition, the figure shows that the H2A/H2B ratio slightly increases as H2A.Z decreases. This clearly shows that the depletion is specific for H2A.Z. Quite strikingly, Figure 3D also shows that H2A.Z re-appears on the body of genes during or after repression (compare 15 minutes with 2 hours) suggesting that the variant histone is actively loaded within chromatin. We therefore propose that an active mechanism allows for non-targeted/random H2A.Z incorporation in the genome and that transcription antagonizes this recruitment.

### Underacetylated H2A.Z accumulates in heterochromatic regions

**Figure 4:**
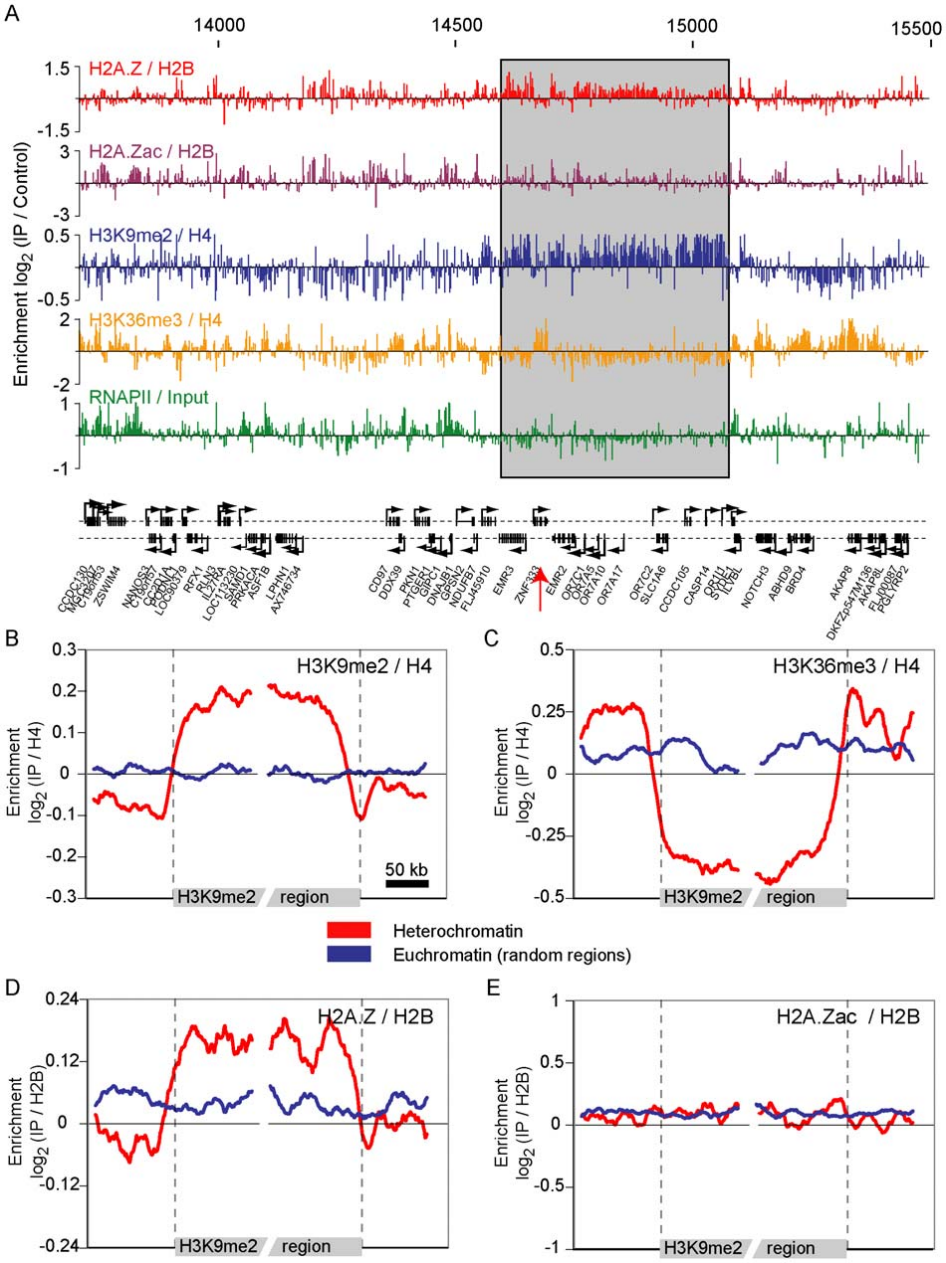
Hypo-acetylated H2A.Z is a marker of facultative heterochromatin. (A) H2A.Z (red), acetylated-H2A.Z (purple), H3K9me2 (blue), H3K36me3 (gold), and RNAPII (green) enrichments are shown over a portion of chromosome 19 that contains a heterochromatic region (Boxed in grey; defined as a region with high level of H3K9me2; see Materials and Methods). The *ZNF333* gene that is free of heterochromatic features is identified by a red arrow. (B–E) Mapping of the average enrichment signal of H3K9me2 (B), H3K36me3 (C), H2A.Z (D), and acetylated-H2A.Z (E) on heterochromatic regions (red) and randomly selected euchromatic regions (blue). The coordinates of all the heterochromatin regions identified in this study are available in Dataset S7.

A prediction of the “transcription-dependent eviction model” proposed here is that genes that have not been transcribed for a long time should accumulate “abnormally” high levels of H2A.Z on their transcribed region. The best cases for such long-term-repressed genes lie within heterochromatin. Quite strikingly, and in agreement with the model, high levels of H2A.Z are associated with heterochromatic regions (Figure 4). Figure 4A shows an example of such a heterochromatic region (defined here as a region with high level of histone H3K9me2), while Figure 4B–4E shows the average signal of H3K9me2, H3K36me3, H2A.Z and acetylated-H2A.Z over a composite of the 30 heterochromatic regions we have identified on chromosome 19. Heterochromatin-associated H2A.Z is hypo-acetylated relative to euchromatin-associated H2A.Z (Figure 4A and 4E), and is not limited to promoter regions but rather covers transcribed regions (Figure 4D and Figure S11). This further suggests that H2A.Z accumulates in heterochromatin as a consequence of a lack of transcription. Also noteworthy is the fact that heterochromatic genes that manage to escape silencing (that is that they have low H3K9me2 and high H3K36me3) do not show over-accumulation of H2A.Z on their gene body (see red arrow in Figure 4A and Figure S11). Taken together, this data suggests that H2A.Z accumulates on the body of non-transcribed genes, and that this may play a role in shaping heterochromatin.

## Discussion

**Figure 5:**
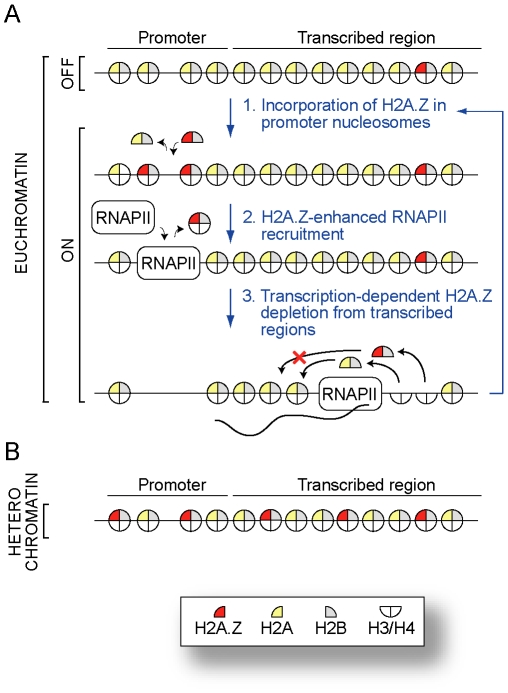
A model illustrating the role of H2A.Z in promoting RNAPII recruitment at promoter and the role of RNAPII in depleting H2A.Z from transcribed regions. (A) H2A.Z in euchromatin. Euchromatic genes that are not transcribed (OFF) do not carry H2A.Z on their promoter. Like for any genomic region, some random nucleosomes may be decorated by the presence of H2A.Z (red). At euchromatic genes that are transcribed, a cycle of H2A.Z recruitment followed by H2A.Z-enhanced RNAPII recruitment is observed at the promoter. In the transcribed region, transcription promotes the depletion of randomly incorporated H2A.Z. The exact mechanism by which this depletion occurs remains unknown but we have depicted what we currently consider to be the most likely option, that is: dimers are removed in the front of the elongating polymerase and reassembled in the wake of RNAPII as previously established [41]. We propose that dimer reassembly is biased toward H2A-containing dimers, leading to a depletion of H2A.Z from transcribed regions. (B) H2A.Z in heterochromatin. In heterochromatin, H2A.Z covers large regions encompassing several genes. Several H2A.Z molecules are therefore associated with heterochromatic genes. It is possible that the absence of transcription in these regions leads to this accumulation of H2A.Z via the random incorporation pathway. Alternatively a mechanism that specifically targets H2A.Z to heterochromatin may exist.

Previous studies have demonstrated the importance of H2A.Z in transcription, DNA repair and genome stability. Here we provide a strong case for a rather global role of H2A.Z in mediating gene expression and demonstrate that the variant histone acts early during transcription, assisting in RNAPII recruitment. In addition, we provide evidence that H2A.Z is randomly incorporated into the genome at low level and that transcription has an antagonizing effect on this random H2A.Z incorporation. This battle between random H2A.Z incorporation and transcription-dependent H2A.Z depletion helps shaping the euchromatin and heterochromatin landscapes. This is summarized in a model shown in Figure 5.

### Human H2A.Z is recruited to promoters “as needed”

Our genomic data shows that human H2A.Z associates with promoters occupied by RNAPII. Time course experiments, however, showed that H2A.Z and RNAPII do not co-occupy the promoter since H2A.Z is recruited prior to RNAPII and is evicted as the polymerase is loaded. Human H2A.Z is therefore recruited to promoters as part of the activation process; which is different from what is observed in yeast, where the association of Htz1 to promoters does not require any activation cues. Htz1 is rather pre-bound to most promoters and it has been proposed that its loading actually occurs during transcriptional repression in order to prime the promoter for the next activation cycle [42]. The reason why mammalian cells have evolved ways to recruit H2A.Z “on demand” may reflect the fact that a small fraction of mammalian genes are actively transcribed at a given time. Yeasts, on the other hand, express a large fraction of their genome at any time and may therefore benefit from having most of their genes primed with Htz1 at all time. Human enhancers, however, are more like yeast core promoters in that they are occupied by H2A.Z regardless of the transcriptional statue of their associated gene (see Figure S2).

### H2A.Z contributes to gene poising by recruiting RNAPII

While we see transient recruitment of H2A.Z upon activation of inducible genes such as *IL8* and *CCL2*, we also observe large amount of H2A.Z on promoters carrying paused RNAPII but no evidence for transcription. At these promoters, the ultimate activation cue (the one that would stimulate the release RNAPII from the pause site and its entry into elongation) is not present. Hence, the presence of H2A.Z at promoters does not require full transcriptional activity. We rather propose that H2A.Z is recruited upon transacting signals that triggers RNAPII recruitment. At some promoters, such a signal is sufficient to activate transcription. This is the case at *IL8*, where we observe H2A.Z-dependent recruitment of RNAPII and active transcription upon dauno treatment. At *CCL2*, however, a significant amount of RNAPII is pre-bound to the promoter in the absence of dauno. Upon dauno treatment, we observe release of RNAPII into elongation and subsequent association of H2A.Z followed by re-association of RNAPII to the promoter. We therefore conclude that transient association of H2A.Z with promoters always precedes (and favors) RNAPII recruitment; and that this may lead to transactivation or promoter poising depending on the promoter context. Interestingly, H2A.Z reappears on the promoter after the first wave of RNAPII has left the promoter, suggesting that H2A.Z is not only recruited during the initial round of transcription but is reloaded prior to each new round. This suggests that the presence of H2A.Z on the promoter may play an active role beyond that of creating unstable promoter chromatin.

### Recruitment of RNAPII: a universal function of H2A.Z

Our data show that the transient association of H2A.Z with human promoters helps with the recruitment of RNAPII. We observed the same in yeast [27], suggesting that the function of the variant histone at promoters is conserved, although the strategy used to recruit it may be different between yeast and mammals. Many mechanisms have been proposed to explain how Htz1 may regulate transcription in yeast (see Introduction). The knowledge that mammalian cells also use H2A.Z to help recruiting RNAPII to promoters will prompt us and others to test these mechanisms in higher eukaryotes as well. Sequence analysis of H2A.Z-bound promoters showed that these are enriched in CpG-island (data not shown). Since H2A.Z has been shown to antagonize DNA methylation in *Arabidopsis* [23], it is possible that CpG-rich promoters use the presence of H2A.Z to counteract DNA methylation–dependent silencing.

### Elongating RNAPII can discriminate between H2A molecules

Perhaps the most striking finding in this study is the fact that transcription results in the removal H2A.Z from transcribed regions. Transcriptional elongation was known to perturb chromatin structure but, to our knowledge, this study represents the first evidence that the elongation complex can discriminate between canonical histones and their variants. Most likely, elongation disrupts both H2A- and H2A.Z-containing dimers but a transcription-coupled mechanism would prevent H2A.Z-dimers to re-associate in the wake of RNAPII. What allows elongation complexes to discriminate between histone molecules is unknown but may be linked to chromatin assembly/disassembly complexes as well as the various histone chaperones that interact with the elongating polymerase. Activities such as FACT, Asf1, Rtt106, Spt6, Spt2, Chd1 and others have been shown or proposed to be important for the co-transcription chromatin assembly [41], [43]–[46]. A preference of those machines for canonical H2A would make the transcription-dependent H2A.Z depletion possible. Also interesting, is the possibility that H2A.Z depletion may be linked to the well established incorporation H3.3 in transcribed regions [47]. Indeed, H3.3/H2A.Z nucleosomes are known to be relatively unstable [48],[49]. It is therefore tempting to speculate that H2A.Z depletion may be coupled to H3.3 deposition. Alternatively, it is possible that the depletion of H2A.Z is required for the incorporation of H3.3.

The question of why cells have evolved mechanisms to remove H2A.Z during elongation will require additional work, but we envision that it may play a role in repressing cryptic initiation. Indeed, the fact that the presence of H2A.Z within promoters helps recruiting RNAPII suggests that removing H2A.Z molecules from transcribed region may be important to suppress cryptic initiation inside genes. Alternatively, removing H2A.Z from transcribed region may somehow help with subsequent transcription rounds, a model compatible with previous work suggesting that this variant histone affects elongation [30].

Also striking is the fact that H2A.Z molecules quickly re-appear on genes after transcription has ceased. Indeed, the level of H2A.Z molecule in recently transcribed genes reaches background level within 2 hours after cessation of transcription. This implies that random H2A.Z incorporation occurs via an active mechanism. Whether this non-targeted incorporation proceeds via SWR1 (in yeast), p400 and SRCAP (mammals) like for promoter-targeted incorporation will require additional experiments.

### Different “flavors” of H2A.Z in promoters and in heterochromatin

The H2A.Z molecules associated with promoters are acetylated while the ones that accumulate in heterochromatin are under-acetylated. Elegant immunostaining work by the Chueng laboratory showed that a ubiquitinylated form of H2A.Z associates with the inactive X chromosome [34]. It is not clear at this point whether the non-acetylated H2A.Z found in facultative heterochromatin is also ubiquitinylated but this possibility is attractive. It will be interesting to test if a cross-talk exists between H2A.Z acetylation (or deacetylation) and ubiquitination.

### The role of H2A.Z in heterochromatin

It is tempting to speculate that the accumulation of under-acetylated H2A.Z in facultative heterochromatin plays a role in gene silencing. So far, H2A.Z was shown to be important for the formation of pericentromeric heterochromatin [50] but its role in the function of facultative heterochromatin remains unexplored. Using H2A.Z-specific shRNA, we were not able to show any defect in the expression of heterochromatic genes in U2OS cells (data not shown). This data suggests that the presence of H2A.Z in heterochromatic regions is not required for maintaining silencing. This could mean that H2A.Z accumulation in heterochromatin is solely a bi-product of these genes not being transcribed. Alternatively, H2A.Z accumulation may be important for the *de novo* formation of heterochromatin rather than its maintenance. Finally, it remains possible that H2A.Z plays a role in other aspects of heterochromatin function. All these possibilities will require further experimentations but the data shown here clearly establishes the accumulation of hypo-acetylated H2A.Z in large domains as a landmark of facultative heterochromatin.

### Mammalian H2A.Z does not restrict the spreading of heterochromatin

In yeast, Htz1 was shown to restrict the spreading of Sir2-dependent silencing [51]. Despite the fact that Sir2-dependent silencing does not have its equivalent in mammals, the presence of H2A.Z in heterochromatin suggested that it might play as role in restraining heterochromatin spreading. Investigation of H3K9me2 profiles in H2A.Z knockdown experiments did not allow detecting any sign of heterochromatin spreading (not shown). This result is not very surprising since mammalian heterochromatin is very different than Sir2-dependent “heterochromatin” and probably uses different spreading mechanisms.

The work presented here provides clear evidence that H2A.Z is recruited to chromatin via at least two distinct mechanisms. First, a very dynamic recruitment of H2A.Z at promoters plays a role in the recruitment of RNAPII. Second, a non-targeted mechanism allows for the random incorporation of some H2A.Z molecules in the genome. Finally, we show that a process linked to transcriptional elongation allows for the depletion of the variant histone from active genes. This battle between active H2A.Z incorporation and transcription-dependent H2A.Z depletion shapes the euchromatin and heterochromatin landscape of mammalian cells. Because heterochromatin formation plays a key role in normal development and in tumorigenesis, the results presented here shall open the way to experiments aiming at better understanding the role of H2A.Z in these important aspects of biology.

## Materials and Methods

### Cell culture and treatment

U2OS cells were grown in McCoy's medium in the presence of 10% FBS, 0.2 mM L-glutamine, 100 IU/mL penicillin and 100 µg/mL streptomycin at 37°C in the presence of 5% CO_2_. For time course experiments, cells were serum-deprived (by changing the medium to 0.5% FBS) for 24 hours prior to addition of 250 nM dauno for various periods of time as indicated in figure legends. ShRNA-mediated knockdowns were performed as described previously [24]. Yeast cells (yFR212: MATa, ade2-1, trp1-1, can1-100, leu2-3,112, his3-11,15, ura3-1, HTZ1::3myc; yFR134: MATa, ade2-1, trp1-1, can1-100, leu2-3,112, his3-11,15, ura3, HTA1::9myc::TRP1) carrying respectively three and nine myc epitopes in the C-terminal end of endogenous HTZ1 or HTA1 were used in ChIP-chip experiments. Heat shock was performed as follow. Cells were grown in YPD at 25°C until mid-log phase and an aliquot (to be used as t0) was taken prior to adding an equal volume of 49°C YPD medium (to instantly raise the temperature to 37°C). The cells were put at 37°C and an aliquot was taken at 15 minutes. Cells were then put back at 25°C and a last aliquot was taken at 120 minutes. All aliquots were used in ChIP as described below.

### Chromatin immunoprecipitations

For mammalian cells, chromatin immunoprecipitations were performed as described previously [24] starting from 10^8^ U2OS cells. For yeast cells, chromatin immunoprecipitations were performed as described previously [15]. The antibodies used were as follow: H2A.Z (Ab4174), acetylated-H2A.Z (Ab18262), H3K9me2 (Ab1220), H3K36me3 (Ab9050), H3K4me3 (Ab8580), H3K4me1 (Ab8895), RNAPII (8WG16), H2B (Ab1790), recombinant H4 (a gift from Alain Verreault), Myc-tagged Htz1and Hta1 in yeast ChIPs (9E10).

### Labeling and hybridization

DNA from mammalian or yeast ChIP and controls samples were labeled using a double or single amplification protocol respectively as described previously [15],[52].

### Normalization

The data were normalized using limma's loess function [53] in BioConductor (from the ArrayPipe Analysis Pipeline (ref PMID: 15215429) and replicates were combined using a weighted average method as described previously [54]. The combined datasets are available in Dataset S1, S2, S3, S4, S5, S6.

### Mapping data to average genes

To interpolate between probes, a standard Gaussian filter (SD = 200 bp) was applied twice to the data as described previously [15] to generate a value each 10 bp. This “smoothed data” was used to calculate the average signal in the promoter (defined as TSS±500 bp) and on the complete gene length of each gene (using the UCSC_knownGenes_hg18 from Mar. 2008). Genes (using unique TSS) were then binned into groups as described in the figure legend. The non-smoothed data was mapped on the 5′ and 3′ boundaries into 200 bp windows for each half-gene and adjacent half-intergenic regions. A sliding window of 1.6 kb was then applied to the ratios. The same mapping procedure was used to map the yeast Htz1 dataset (Figure S10) using 50 bp windows and a sliding window of 300 bp.

### Mapping data to average K9me2 regions

(Figure 4B–4E) To identify heterochromatic regions, the combined H3K9me2 dataset was smoothed as described above, except that the standard deviation was 10 kb instead of 200 bp. Regions of interest were defined as consecutive positions above 0 covering at least 50 kb, with an average intensity of at least 0.1. Regions separated by less than 50 kb were then merged, identifying 30 “heterochromatic regions” on chromosome 19. The non-smoothed data was mapped on the 5′ and 3′ boundaries into 1 kb windows for each half-region and a sliding window of 20 kb was then applied to the ratios.

### Mapping data to average K4me1 regions

(Figure S2) To identify predicted enhancers, we applied a strategy developed by the Ren laboratory [33]. Regions specifically enriched in H3K4me1 (p<0.0.1) were identified using an algorithm described previously [52]. The regions were then filtered to include only those located with intergenic regions and that are at least 5 kb from a known TSS (to avoid contamination from proximal promoters). This led to the identification of 577 predicted enhancers. The middle of each region was used to map the different non-smoothed data into 50 bp windows and a sliding window of 1.5 kb was then applied to the ratios. Enhancers were then classified in three groups based on the transcriptional status of the closest TSS within the same chromosomal domain as defined by a regions flanked by two CTCF binding sites [55].

### RNAPII and H2A.Z occupancy at yeast genes

(Figure 3D) The data was smoothed as described above (SD = 200 bp, resolution = 10 bp) and the average signal was calculated in the middle of each protein-coding gene (using SGD version Feb. 02 2008; http://www.yeastgenome.org/) in a window excluding the first and last 250 pb to avoid contamination by intergenic signal (genes<500 bp were then discarded).

## Supporting Information

Figure S1 The average signal of most histone marks and proteins tested in this study on the 2439 unique TSS of chr19. H2A.Z/H2B (red), RNAPII/input (green), P-Ser2 RNAPII/input (grey), H3K36me3/H4 (gold), H3K4me3/H4 (dark blue), H3K4me1/H4 (light blue), and H4/input (yellow).
(0.39 MB PDF)

Figure S2 H2A.Z occupies distal regulatory elements. (A) Distal regulatory elements were defined as statistically enriched H3K4me1regions in intergenic regions (based on [1]) that are >5 kb from a known TSS. H3K4me1 (light blue), H3K4me3 (dark blue), RNAPII (green), and H2A.Z (red) were mapped on these 577 regions. The dotted lines represent mapping on randomly selected regions. (B) H2A.Z is present both on active and inactive distal regulatory elements. Distal regulatory elements (as defined in panel A) were classified in three groups based on the transcriptional status of the closest TSS within the same chromosomal domain (defined using CTCF binding sites [2]). Inactive enhancers (260, blue) are defined as those for which the closest gene is free of RNAPII. Poised enhancers (155, green) were defined as those for which the closest gene has paused (non-processive) RNAPII (presence of RNAPII but absence of H3K36me3). Active enhancers (110, gold) were defined as those for which the closest gene has processive RNAPII (presence of both RNAPII and H3K36me3). The red curve shows all enhancers as in panel A. (1. Heintzman ND, Stuart RK, Hon G, Fu Y, Ching CW et al. (2007) Distinct and predictive chromatin signatures of transcriptional promoters and enhancers in the human genome. Nat Genet 39: 311–318. 2. Kim TH, Abdullaev ZK, Smith AD, Ching KA, Loukinov DI et al. (2007) Analysis of the vertebrate insulator protein CTCF-binding sites in the human genome. Cell 128: 1231–1245.)
(0.48 MB PDF)

Figure S3 A complement to Figure 1A–1B. (A) The average RNAPII occupancy calculated on each promoter is shown. Genes were binned into 6 different categories from high RNAPII occupancy (red) to low RNAPII occupancy (purple) (166, 346, 351, 766, 634, and 176 genes respectively) and used in Figure 1B and 1C. (B) Scatter plot of the average RNAPII and H2A.Z enrichment ratios observed over promoters. (C–H) Mapping of acetylated H2A.Z/H2B (C), H3K4me3/H4 (D), H3K36me3/H4 (E), H3K4me1/H4 (F), P-Ser2 RNAPII/input (G), and H4/input (H) on gene groups defined in panel A.
(1.61 MB PDF)

Figure S4 Same analysis as for Figure 1D–1E but using H3K36me3 levels (instead of P-Ser2 RNAPII) to estimate transcription rate. (A) The average P-Ser2 RNAPII enrichment calculated on genes is shown. Only the genes with high levels of RNAPII were used (the union of the red, orange and yellow groups of Figure S3). Genes were binned into 4 different categories from high H3K36me3 (red) to low H3K36me3 (blue) (228, 350, 126, and 159 genes respectively) and used in panels B–H. (B–H) Mapping of H3K36me3 (B), acetylated H2A.Z/H2B (C), H2A.Z/H2B (D), H3K9me2/H4 (E), RNAPII/input (F), P-Ser2 RNAPII/input (G) and, H3K4me3/H4 on groups defined in panel A.
(1.12 MB PDF)

Figure S5 A complement to Figure 1D–1E (A) The average P-Ser2 RNAPII enrichment calculated on genes is shown. Only the genes with high levels of RNAPII were used (The union of the red, orange and yellow groups of Figure S3). Genes were binned into 4 different categories from high P-Ser2 RNAPII (red) to low P-Ser2 RNAPII (blue) (215, 253, 258, and 137 genes respectively) and used in Figure 1D and 1E. (B) Scatter plot of the average P-Ser2 RNAPII and H3K36me3/H4 enrichment ratios observed over genes. (C–H), Mapping of acetylated H2A.Z/H2B (C), RNAPII/input (D), H3K9me2/H4 (E), H3K4me3/H4 (F), H3K36me3/H4 (G) and H3K4me1/H4 (H) on gene groups defined in panel A.
(0.73 MB PDF)

Figure S6 Gene expression profiling of daunorubicine treatment. U2OS cells were treated with dauno as described in Materials and Methods. Expression profiles were determined using the Illumina microarray platform. A scatter plot of the log2 expression signal is shown for the 0 minute vs. 240 minutes after dauno treatment. The *IL8* and *CCL2* genes are indicated.
(0.29 MB PDF)

Figure S7 Additional replicates to the experiment shown in Figure 1F. RNAPII and H2A.Z enrichment are shown over time after dauno treatment in serum-deprived (G1/G0-arrested) cells on the promoter of IL8 (left) and CCL2 (right).
(0.29 MB PDF)

Figure S8 A complement to Figure 2A–2B. (A) Knockdown of H2A.Z cripples the activation of IL8 upon dauno treatment. Data from individual replicates of the experiment shown in Figure 2A. (B) Knockdown of H2A.Z cripples the recruitment of RNAPII to the IL8 promoter upon dauno treatment. Data from individual replicates of the experiment shown in Figure 2B.
(0.26 MB PDF)

Figure S9 A complement to Figure 2C. Knockdown of H2A.Z causes a general decrease of RNAPII on genes as opposed to a major reshuffling of the transcriptome. RNAPII enrichment from cells treated with control shRNAs is plotted against the enrichment of RNAPII from cells treated with H2A.Z-specific shRNAs.The red line shows the trend of the data while the green line represent a slope of 1, corresponding to what would have bend expected with no global effect of H2A.Z. A massive reshuffling, on the other hand, would have generated no correlation.
(0.41 MB PDF)

Figure S10 H2A.Z is depleted from transcribed open reading frames in yeast. Yeast H2A.Z ChIP-chip data from [1] were mapped on gene groups based on expression level based on [2]: The red group contains the most transcribed genes while the blue group contains the least transcribed yeast genes (662, 2964, 1345, and 1053 genes respectively). (1. Guillemette B, Bataille AR, Gevry N, Adam M, Blanchette M et al. (2005) Variant histone H2A.Z is globally localized to the promoters of inactive yeast genes and regulates nucleosome positioning. PLoS Biol 3: e384. 2. Holstege FC, Jennings EG, Wyrick JJ, Lee TI, Hengartner CJ et al. (1998) Dissecting the regulatory circuitry of a eukaryotic genome. Cell 95: 717–728.)
(0.23 MB PDF)

Figure S11 H2A.Z covers the entire length of heterochromatic genes except for genes that escape silencing. (A) Genome browser screenshots of two heterochromatic regions on chromosome 19 that contain one gene that escapes silencing (red arrows). (B–G) The average ChIP-chip signal is shown over all unique TSS inside heterochromatic regions (392 genes, red), euchromatic genes (392 genes, blue), and heterochromatic genes that escaped silencing (50 genes, green). The genes that escape silencing are defined as genes with low H3K9me2 and high H3K36me3 that are contained within a heterochromatic region. The data are shown for H2A.Z/H2B (B), acetylated H2A.Z/H2B (C), RNAPII/input (D), H3K36me3/H4 (E), H3K4me3/H4 (F), and H3K9me2/H4 (G).
(1.08 MB PDF)

Figure S12 Removing the ZNF genes from our dataset does not affect the results shown in Figure 1. Because chromosome 19 contains a certain number of ZNF genes–and because these genes were shown to sometimes have a peculiar chromatin structure–we wished to repeat the analyses shown in Figure 1B–1E by first removing all ZNF genes from the dataset. Panels A, B, C, and D represent mirror analyses of panels B, C, D, and E from Figure 1 respectively, except that all ZNF genes were remove before computing the data.
(0.80 MB PDF)

Table S1 Table containing the list of all experiments described in this work.
(0.05 MB DOC)

Dataset S1 UCSC Genome browser-ready file containing the log2 enrichment ratios for the RNAPII/input, H2A.Z/H2B, H2A.Z/input and H2A.Z/H4 ChIP-chip experiments. The sum of Dataset S1, S2, S3, S4, S5 represents all the human ChIP-chip experiments described in this work.
(8.49 MB GZ)

Dataset S2 UCSC Genome browser-ready file containing the log2 enrichment ratios of the acH2A.Z/H2B, H2B/H4, and RNAPII P-Ser2/input ChIP-chip experiments described in this work. The sum of Dataset S1, S2, S3, S4, S5 represents all the human ChIP-chip experiments described in this work.
(7.82 MB GZ)

Dataset S3 UCSC Genome browser-ready file containing the log2 enrichment ratios of the H3K36me3/H4 and H4K4me1/H4 ChIP-chip experiments described in this work. The sum of Dataset S1, S2, S3, S4, S5 represents all the human ChIP-chip experiments described in this work.
(6.32 MB GZ)

Dataset S4 UCSC Genome browser-ready file containing the log2 enrichment ratios of the H3K4me2/H4, H4K4me3/H4, and H3K9me2/H4 ChIP-chip experiments described in this work. The sum of Dataset S1, S2, S3, S4, S5 represents all the human ChIP-chip experiments described in this work.
(8.50 MB GZ)

Dataset S5 UCSC Genome browser-ready file containing the log2 enrichment ratios of the H4/input, RNAPII in shH2A.Z, and RNAPII in shControl ChIP-chip experiments described in this work. The sum of Dataset S1, S2, S3, S4, S5 represents all the human ChIP-chip experiments described in this work.
(6.99 MB GZ)

Dataset S6 UCSC Genome browser-ready file containing the log2 enrichment ratios of all yeast ChIP-chip experiments described in this work.
(3.91 MB GZ)

Dataset S7 UCSC Genome browser-ready file containing the heterochromatin regions identified in this study using H3K9me2.
(0.00 MB GZ)

## References

[1] Jin J, Cai Y, Li B, Conaway RC, Workman JL (2005). In and out: histone variant exchange in chromatin.. Trends Biochem Sci.

[2] Zlatanova J, Thakar A (2008). H2A.Z: view from the top.. Structure.

[3] Guillemette B, Gaudreau L (2006). Reuniting the contrasting functions of H2A.Z.. Biochem Cell Biol.

[4] Suto RK, Clarkson MJ, Tremethick DJ, Luger K (2000). Crystal structure of a nucleosome core particle containing the variant histone H2A.Z.. Nat Struct Biol.

[5] Liu X, Li B, GorovskyMa (1996). Essential and nonessential histone H2A variants in Tetrahymena thermophila.. Mol Cell Biol.

[6] Ridgway P, Brown KD, Rangasamy D, Svensson U, Tremethick DJ (2004). Unique residues on the H2A.Z containing nucleosome surface are important for Xenopus laevis development.. J Biol Chem.

[7] Clarkson MJ, Wells JR, Gibson F, Saint R, Tremethick DJ (1999). Regions of variant histone His2AvD required for Drosophila development.. Nature.

[8] Faast R, Thonglairoam V, Schulz TC, Beall J, Wells JR (2001). Histone variant H2A.Z is required for early mammalian development.. Curr Biol.

[9] Dunican DS, McWilliam P, Tighe O, Parle-McDermott A, Croke DT (2002). Gene expression differences between the microsatellite instability (MIN) and chromosomal instability (CIN) phenotypes in colorectal cancer revealed by high-density cDNA array hybridization.. Oncogene.

[10] Rhodes DR, Yu J, Shanker K, Deshpande N, Varambally R (2004). Large-scale meta-analysis of cancer microarray data identifies common transcriptional profiles of neoplastic transformation and progression.. Proc Natl Acad Sci U S A.

[11] Zucchi I, Mento E, Kuznetsov VA, Scotti M, Valsecchi V (2004). Gene expression profiles of epithelial cells microscopically isolated from a breast-invasive ductal carcinoma and a nodal metastasis.. Proc Natl Acad Sci U S A.

[12] Hua S, Kallen CB, Dhar R, Baquero MT, Mason CE (2008). Genomic analysis of estrogen cascade reveals histone variant H2A.Z associated with breast cancer progression.. Mol Syst Biol.

[13] Gevry N, Hardy S, Jacques PE, Laflamme L, Svotelis A (2009). Histone H2A.Z is essential for estrogen receptor signaling.. Genes Dev.

[14] Creyghton MP, Markoulaki S, Levine SS, Hanna J, Lodato MA (2008). H2AZ is enriched at polycomb complex target genes in ES cells and is necessary for lineage commitment.. Cell.

[15] Guillemette B, Bataille AR, Gevry N, Adam M, Blanchette M (2005). Variant histone H2A.Z is globally localized to the promoters of inactive yeast genes and regulates nucleosome positioning.. PLoS Biol.

[16] Raisner RM, Hartley PD, Meneghini MD, Bao MZ, Liu CL (2005). Histone variant H2A.Z marks the 5′ ends of both active and inactive genes in euchromatin.. Cell.

[17] Li B, Pattenden SG, Lee D, Gutierrez J, Chen J (2005). Preferential occupancy of histone variant H2AZ at inactive promoters influences local histone modifications and chromatin remodeling.. Proc Natl Acad Sci U S A.

[18] Millar CB, Xu F, Zhang K, Grunstein M (2006). Acetylation of H2AZ Lys 14 is associated with genome-wide gene activity in yeast.. Genes Dev.

[19] Zhang H, Roberts DN, Cairns BR (2005). Genome-wide dynamics of Htz1, a histone H2A variant that poises repressed/basal promoters for activation through histone loss.. Cell.

[20] Barski A, Cuddapah S, Cui K, Roh TY, Schones DE (2007). High-resolution profiling of histone methylations in the human genome.. Cell.

[21] Mavrich TN, Jiang C, Ioshikhes IP, Li X, Venters BJ (2008). Nucleosome organization in the Drosophila genome.. Nature.

[22] Bruce K, Myers FA, Mantouvalou E, Lefevre P, Greaves I (2005). The replacement histone H2A.Z in a hyperacetylated form is a feature of active genes in the chicken.. Nucleic Acids Res.

[23] Zilberman D, Coleman-Derr D, Ballinger T, Henikoff S (2008). Histone H2A.Z and DNA methylation are mutually antagonistic chromatin marks.. Nature.

[24] Gevry N, Chan HM, Laflamme L, Livingston DM, Gaudreau L (2007). p21 transcription is regulated by differential localization of histone H2A.Z.. Genes Dev.

[25] Albert I, Mavrich TN, Tomsho LP, Qi J, Zanton SJ (2007). Translational and rotational settings of H2A.Z nucleosomes across the Saccharomyces cerevisiae genome.. Nature.

[26] Greaves IK, Rangasamy D, Ridgway P, Tremethick DJ (2007). H2A.Z contributes to the unique 3D structure of the centromere.. Proc Natl Acad Sci U S A.

[27] Adam M, Robert F, Larochelle M, Gaudreau L (2001). H2A.Z is required for global chromatin integrity and for recruitment of RNA polymerase II under specific conditions.. Mol Cell Biol.

[28] Santisteban MS, Kalashnikova T, Smith MM (2000). Histone H2A.Z regulats transcription and is partially redundant with nucleosome remodeling complexes.. Cell.

[29] Larochelle M, Gaudreau L (2003). H2A.Z has a function reminiscent of an activator required for preferential binding to intergenic DNA.. EMBO J.

[30] Farris SD, Rubio ED, Moon JJ, Gombert WM, Nelson BH (2005). Transcription-induced chromatin remodeling at the c-myc gene involves the local exchange of histone H2A.Z.. J Biol Chem.

[31] Brickner DG, Cajigas I, Fondufe-Mittendorf Y, Ahmed S, Lee PC (2007). H2A.Z-mediated localization of genes at the nuclear periphery confers epigenetic memory of previous transcriptional state.. PLoS Biol.

[32] John S, Sabo PJ, Johnson TA, Sung MH, Biddie SC (2008). Interaction of the glucocorticoid receptor with the chromatin landscape.. Mol Cell.

[33] Heintzman ND, Stuart RK, Hon G, Fu Y, Ching CW (2007). Distinct and predictive chromatin signatures of transcriptional promoters and enhancers in the human genome.. Nat Genet.

[34] Sarcinella E, Zuzarte PC, Lau PN, Draker R, Cheung P (2007). Monoubiquitylation of H2A.Z distinguishes its association with euchromatin or facultative heterochromatin.. Mol Cell Biol.

[35] Muse GW, Gilchrist DA, Nechaev S, Shah R, Parker JS (2007). RNA polymerase is poised for activation across the genome.. Nat Genet.

[36] Zeitlinger J, Stark A, Kellis M, Hong JW, Nechaev S (2007). RNA polymerase stalling at developmental control genes in the Drosophila melanogaster embryo.. Nat Genet.

[37] Guenther MG, Levine SS, Boyer LA, Jaenisch R, Young RA (2007). A chromatin landmark and transcription initiation at most promoters in human cells.. Cell.

[38] Egloff S, Murphy S (2008). Cracking the RNA polymerase II CTD code.. Trends Genet.

[39] Seoane J, Le HV, Massague J (2002). Myc suppression of the p21(Cip1) Cdk inhibitor influences the outcome of the p53 response to DNA damage.. Nature.

[40] Lemieux K, Larochelle M, Gaudreau L (2008). Variant histone H2A.Z, but not the HMG proteins Nhp6a/b, is essential for the recruitment of Swi/Snf, Mediator, and SAGA to the yeast GAL1 UAS(G).. Biochem Biophys Res Commun.

[41] Belotserkovskaya R, Oh S, Bondarenko VA, Orphanides G, Studitsky VM (2003). FACT facilitates transcription-dependent nucleosome alteration.. Science.

[42] Gligoris T, Thireos G, Tzamarias D (2007). The Tup1 corepressor directs Htz1 deposition at a specific promoter nucleosome marking the GAL1 gene for rapid activation.. Mol Cell Biol.

[43] Rufiange A, Jacques PE, Bhat W, Robert F, Nourani A (2007). Genome-wide replication-independent histone H3 exchange occurs predominantly at promoters and implicates H3 K56 acetylation and Asf1.. Mol Cell.

[44] Imbeault D, Gamar L, Rufiange A, Paquet E, Nourani A (2008). The Rtt106 histone chaperone is functionally linked to transcription elongation and is involved in the regulation of spurious transcription from cryptic promoters in yeast.. J Biol Chem.

[45] Adkins MW, Tyler JK (2006). Transcriptional activators are dispensable for transcription in the absence of Spt6-mediated chromatin reassembly of promoter regions.. Mol Cell.

[46] Nourani A, Robert F, Winston F (2006). Evidence that Spt2/Sin1, an HMG-like factor, plays roles in transcription elongation, chromatin structure, and genome stability in Saccharomyces cerevisiae.. Mol Cell Biol.

[47] Ahmad K, Henikoff S (2002). The histone variant H3.3 marks active chromatin by replication-independent nucleosome assembly.. Mol Cell.

[48] Jin C, Felsenfeld G (2007). Nucleosome stability mediated by histone variants H3.3 and H2A.Z.. Genes Dev.

[49] Jin C, Zang C, Wei G, Cui K, Peng W (2009). H3.3/H2A.Z double variant-containing nucleosomes mark ‘nucleosome-free regions’ of active promoters and other regulatory regions.. Nat Genet.

[50] Swaminathan J, Baxter EM, Corces VG (2005). The role of histone H2Av variant replacement and histone H4 acetylation in the establishment of Drosophila heterochromatin.. Genes Dev.

[51] Meneghini MD, Wu M, Madhani HD (2003). Conserved Histone Variant H2A.Z Protects Euchromatin from the Ectopic Spread of Silent Heterochromatin.. Cell.

[52] Boyer LA, Lee TI, Cole MF, Johnstone SE, Levine SS (2005). Core transcriptional regulatory circuitry in human embryonic stem cells.. Cell.

[53] Yang YH, Dudoit S, Luu P, Lin DM, Peng V (2002). Normalization for cDNA microarray data: a robust composite method addressing single and multiple slide systematic variation.. Nucleic Acids Res.

[54] Ren B, Robert F, Wyrick JJ, Aparicio O, Jennings EG (2000). Genome-wide location and function of DNA binding proteins.. Science.

[55] Kim TH, Abdullaev ZK, Smith AD, Ching KA, Loukinov DI (2007). Analysis of the vertebrate insulator protein CTCF-binding sites in the human genome.. Cell.

[56] Causton HC, Ren B, Koh SS, Harbison CT, Kanin E (2001). Remodeling of yeast genome expression in response to environmental changes.. Mol Biol Cell.

