# Supplementary figures and images for: The Euchromatic and Heterochromatic Landscapes Are Shaped by Antagonizing Effects of Transcription on H2A.Z Deposition
Source: PLoS Genet. 2009 Oct 16;5(10):e1000687. doi: 10.1371/journal.pgen.1000687 (PMC2754525; doi:10.1371/journal.pgen.1000687)

Figure S1

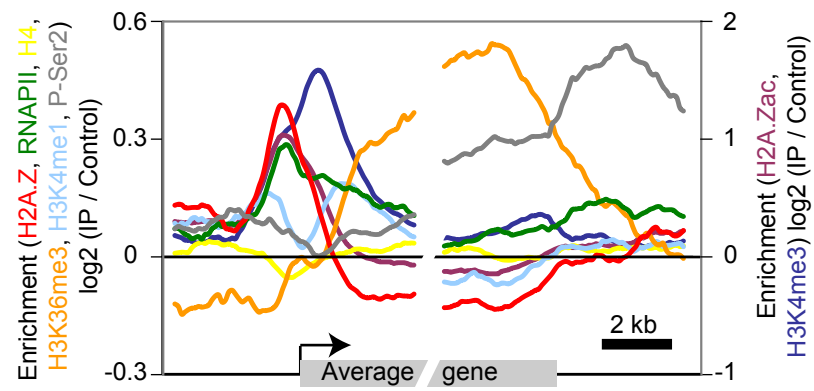

Supplement: Figure S1 — The average signal of most histone marks and proteins tested in this study on the 2439 unique TSS of chr19. H2A.Z/H2B (red), RNAPII/input (green), P-Ser2 RNAPII/input (grey), H3K36me3/H4 (gold), H3K4me3/H4 (dark blue), H3K4me1/H4 (light blue), and H4/input (yellow). (0.39 MB PDF) [file pgen.1000687.s001.pdf]

Figure S2

A

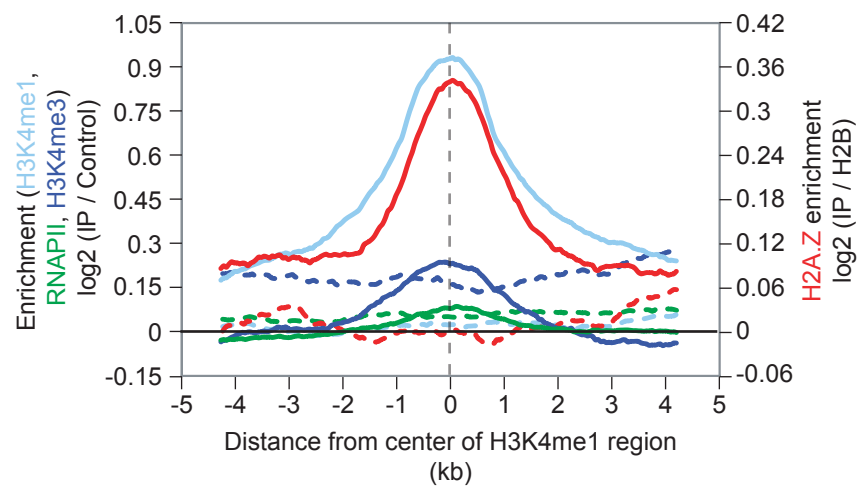

B

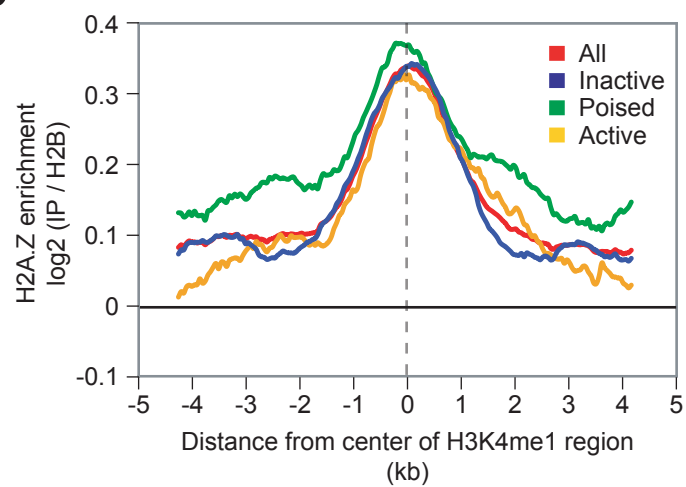

Supplement: Figure S2 — H2A.Z occupies distal regulatory elements. (A) Distal regulatory elements were defined as statistically enriched H3K4me1regions in intergenic regions (based on [1]) that are >5 kb from a known TSS. H3K4me1 (light blue), H3K4me3 (dark blue), RNAPII (green), and H2A.Z (red) were mapped on these 577 regions. The dotted lines represent mapping on randomly selected regions. (B) H2A.Z is present both on active and inactive distal regulatory elements. Distal regulatory elements (as defined in panel A) were classified in three groups based on the transcriptional status of the closest TSS within the same chromosomal domain (defined using CTCF binding sites [2]). Inactive enhancers (260, blue) are defined as those for which the closest gene is free of RNAPII. Poised enhancers (155, green) were defined as those for which the closest gene has paused (non-processive) RNAPII (presence of RNAPII but absence of H3K36me3). Active enhancers (110, gold) were defined as those for which the closest gene has processive RNAPII (presence of both RNAPII and H3K36me3). The red curve shows all enhancers as in panel A. (1. Heintzman ND, Stuart RK, Hon G, Fu Y, Ching CW et al. (2007) Distinct and predictive chromatin signatures of transcriptional promoters and enhancers in the human genome. Nat Genet 39: 311–318. 2. Kim TH, Abdullaev ZK, Smith AD, Ching KA, Loukinov DI et al. (2007) Analysis of the vertebrate insulator protein CTCF-binding sites in the human genome. Cell 128: 1231–1245.) (0.48 MB PDF) [file pgen.1000687.s002.pdf]

# Figure S3

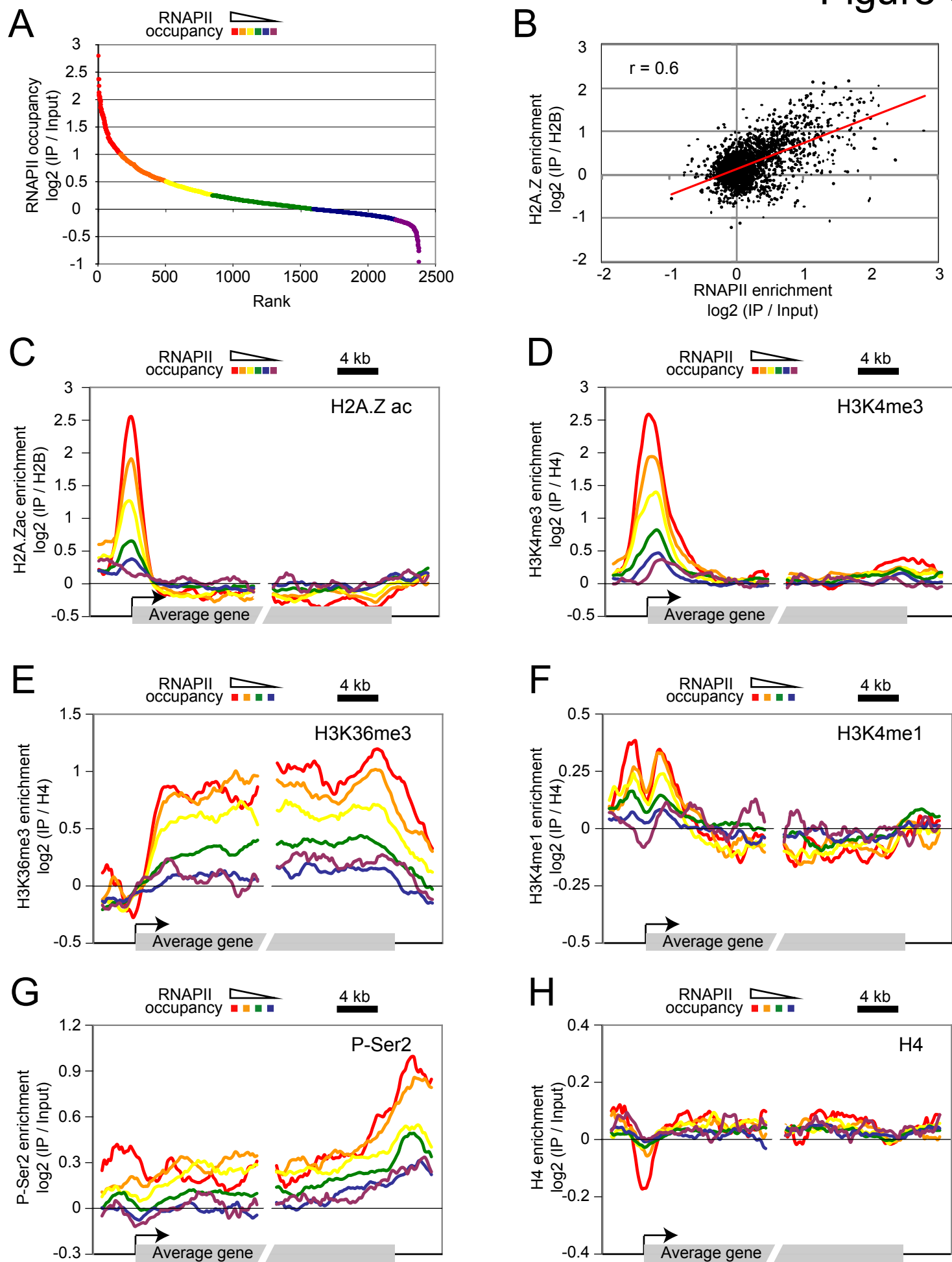

Supplement: Figure S3 — A complement to Figure 1A–1B. (A) The average RNAPII occupancy calculated on each promoter is shown. Genes were binned into 6 different categories from high RNAPII occupancy (red) to low RNAPII occupancy (purple) (166, 346, 351, 766, 634, and 176 genes respectively) and used in Figure 1B and 1C. (B) Scatter plot of the average RNAPII and H2A.Z enrichment ratios observed over promoters. (C–H) Mapping of acetylated H2A.Z/H2B (C), H3K4me3/H4 (D), H3K36me3/H4 (E), H3K4me1/H4 (F), P-Ser2 RNAPII/input (G), and H4/input (H) on gene groups defined in panel A. (1.61 MB PDF) [file pgen.1000687.s003.pdf]

# Figure S4

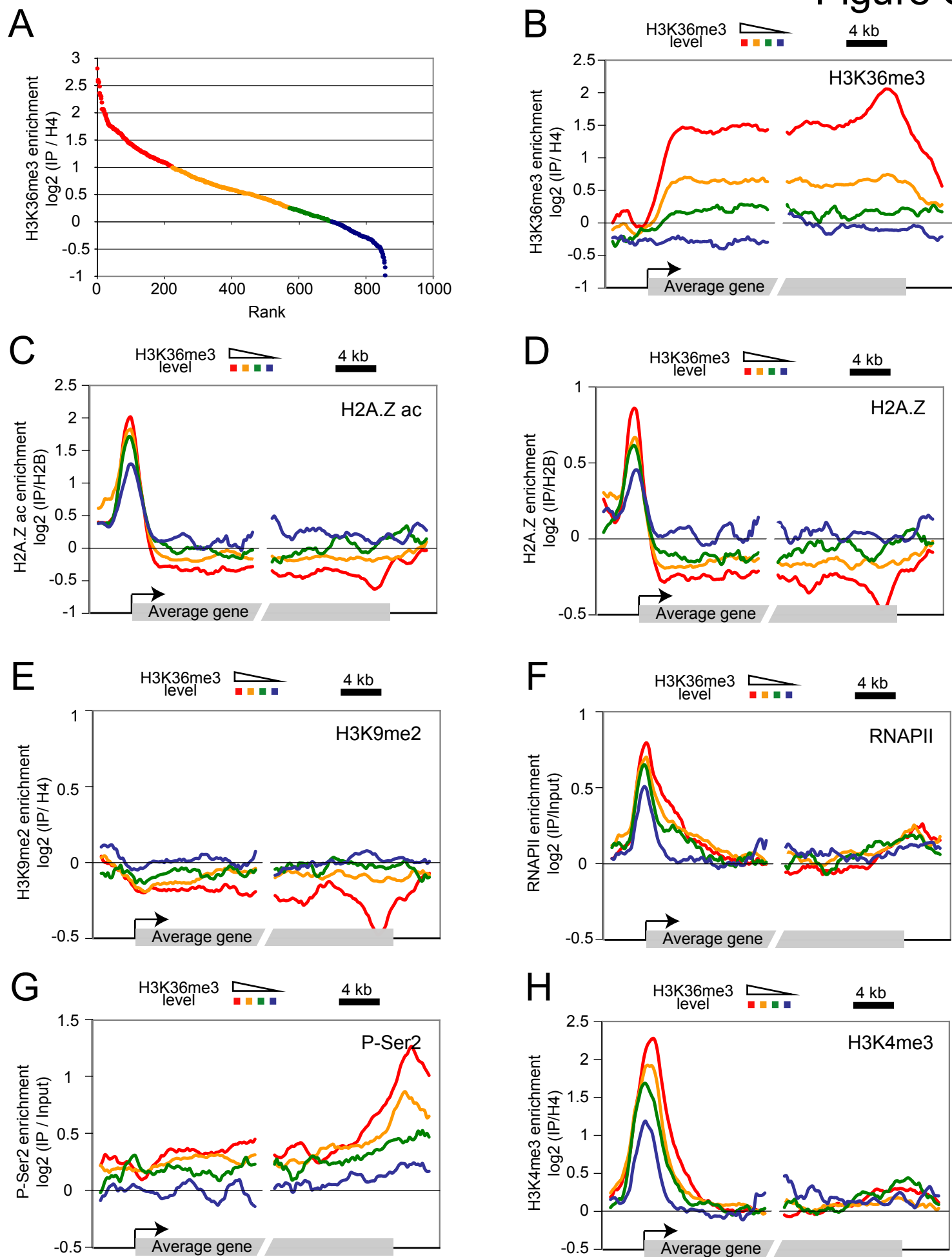

Supplement: Figure S4 — Same analysis as for Figure 1D–1E but using H3K36me3 levels (instead of P-Ser2 RNAPII) to estimate transcription rate. (A) The average P-Ser2 RNAPII enrichment calculated on genes is shown. Only the genes with high levels of RNAPII were used (the union of the red, orange and yellow groups of Figure S3). Genes were binned into 4 different categories from high H3K36me3 (red) to low H3K36me3 (blue) (228, 350, 126, and 159 genes respectively) and used in panels B–H. (B–H) Mapping of H3K36me3 (B), acetylated H2A.Z/H2B (C), H2A.Z/H2B (D), H3K9me2/H4 (E), RNAPII/input (F), P-Ser2 RNAPII/input (G) and, H3K4me3/H4 on groups defined in panel A. (1.12 MB PDF) [file pgen.1000687.s004.pdf]

Figure S5

A

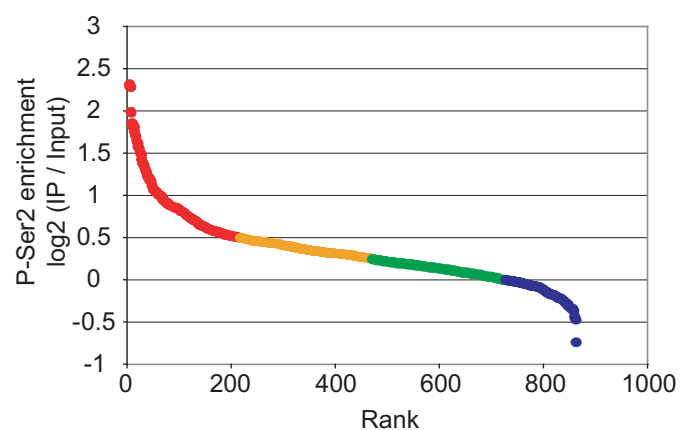

B

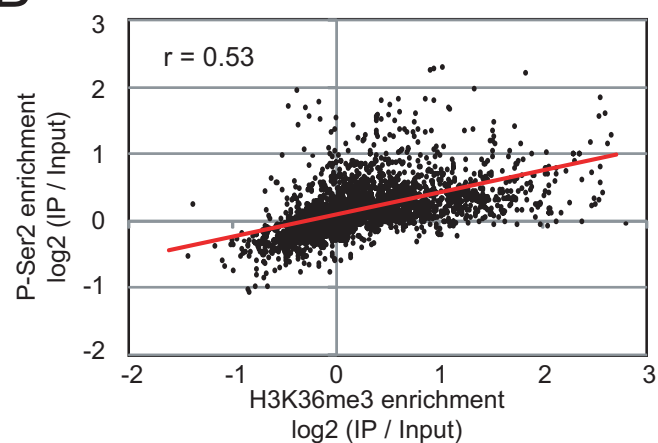

C

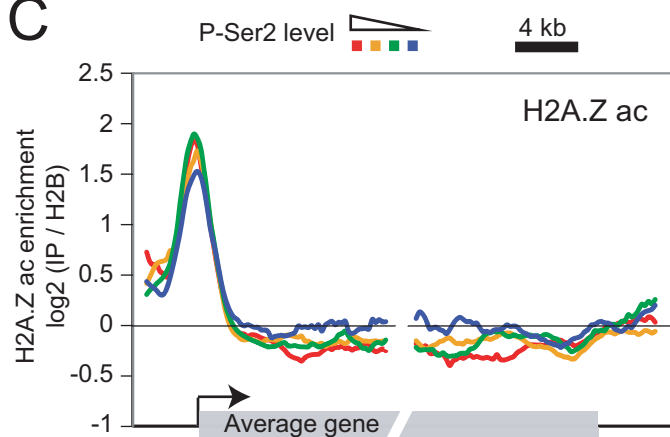

D

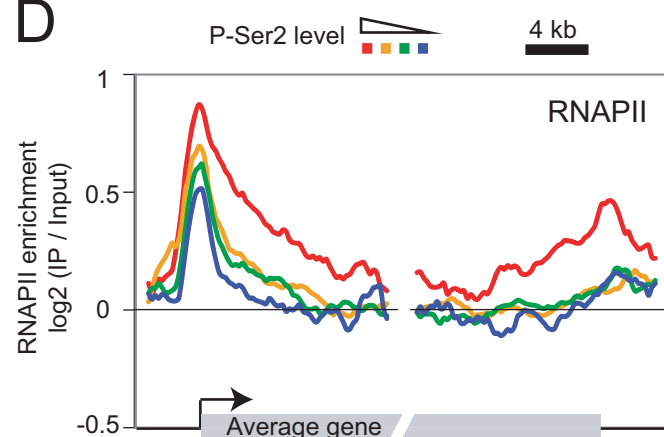

E

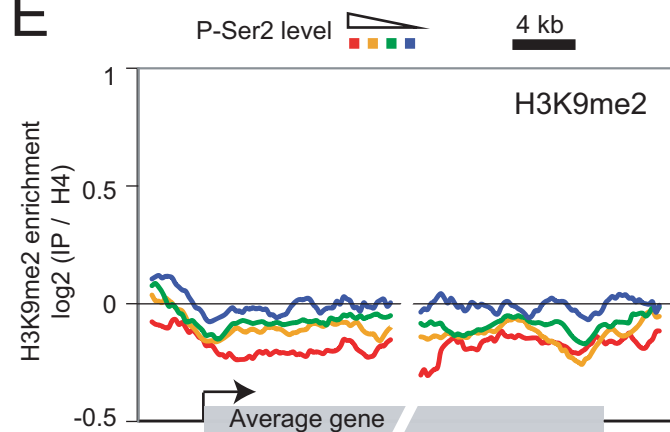

F

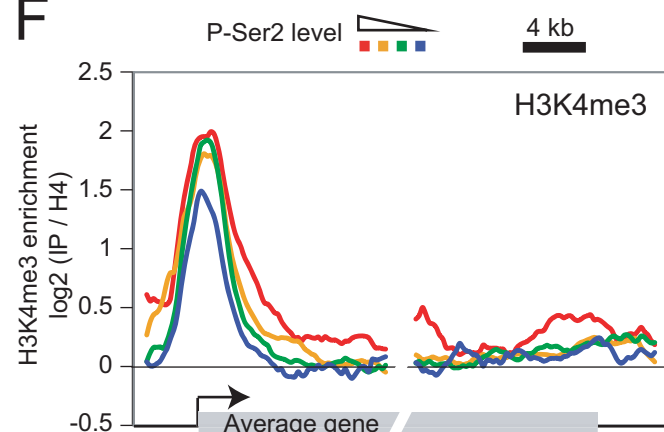

G

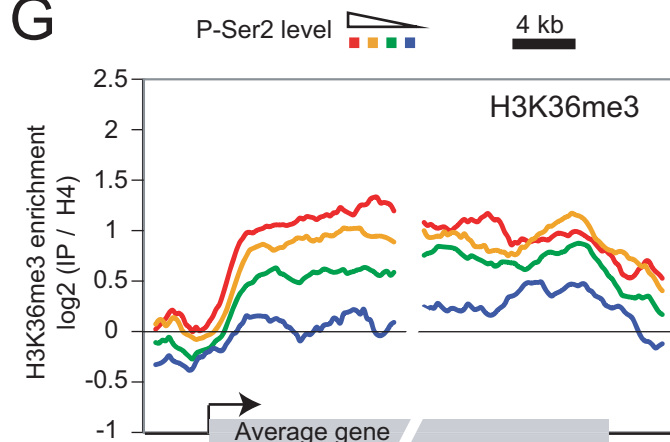

H

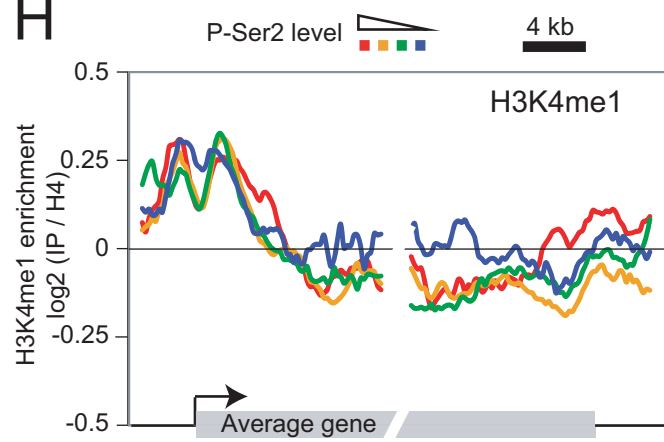

Supplement: Figure S5 — A complement to Figure 1D–1E (A) The average P-Ser2 RNAPII enrichment calculated on genes is shown. Only the genes with high levels of RNAPII were used (The union of the red, orange and yellow groups of Figure S3). Genes were binned into 4 different categories from high P-Ser2 RNAPII (red) to low P-Ser2 RNAPII (blue) (215, 253, 258, and 137 genes respectively) and used in Figure 1D and 1E. (B) Scatter plot of the average P-Ser2 RNAPII and H3K36me3/H4 enrichment ratios observed over genes. (C–H), Mapping of acetylated H2A.Z/H2B (C), RNAPII/input (D), H3K9me2/H4 (E), H3K4me3/H4 (F), H3K36me3/H4 (G) and H3K4me1/H4 (H) on gene groups defined in panel A. (0.73 MB PDF) [file pgen.1000687.s005.pdf]

Figure S6

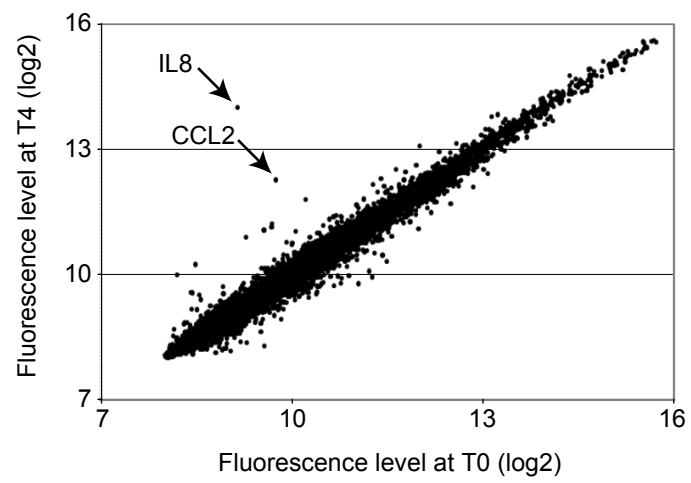

Supplement: Figure S6 — Gene expression profiling of daunorubicine treatment. U2OS cells were treated with dauno as described in Materials and Methods. Expression profiles were determined using the Illumina microarray platform. A scatter plot of the log2 expression signal is shown for the 0 minute vs. 240 minutes after dauno treatment. The IL8 and CCL2 genes are indicated. (0.29 MB PDF) [file pgen.1000687.s006.pdf]

Figure S7

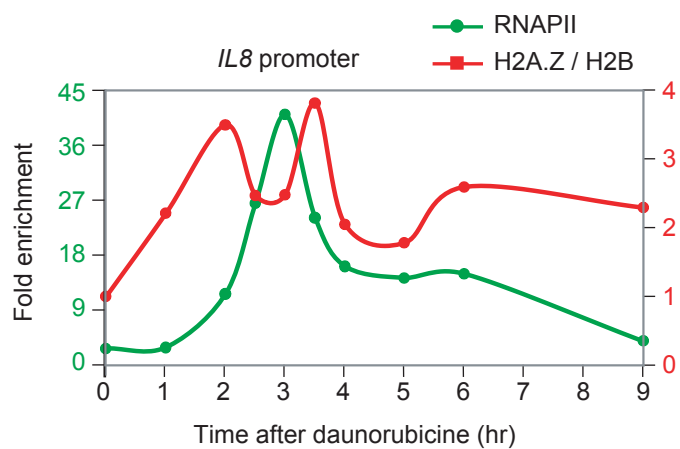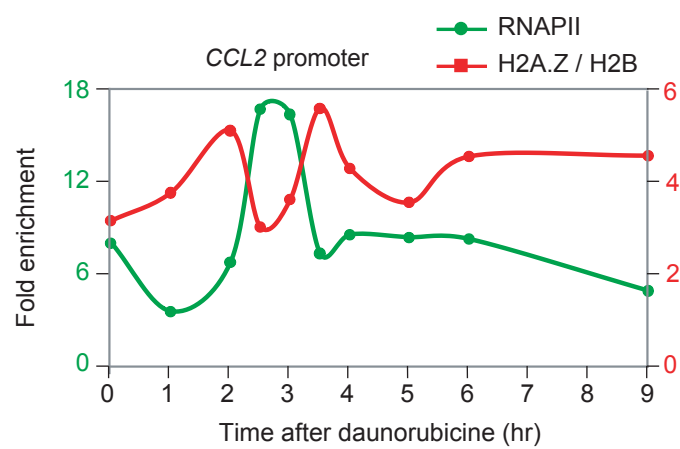

Supplement: Figure S7 — Additional replicates to the experiment shown in Figure 1F. RNAPII and H2A.Z enrichment are shown over time after dauno treatment in serum-deprived (G1/G0-arrested) cells on the promoter of IL8 (left) and CCL2 (right). (0.29 MB PDF) [file pgen.1000687.s007.pdf]

# Figure S8

## A

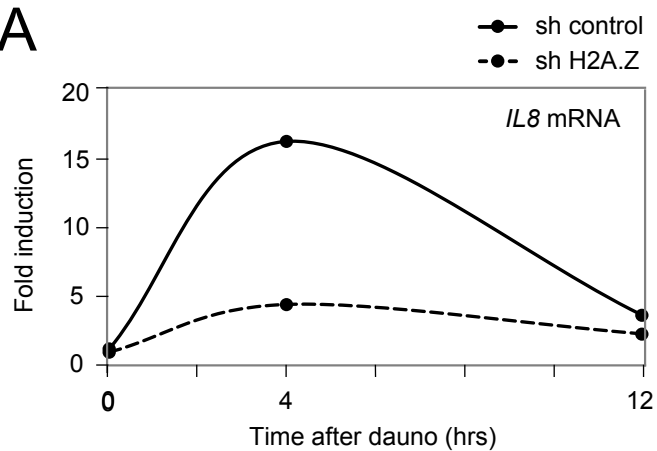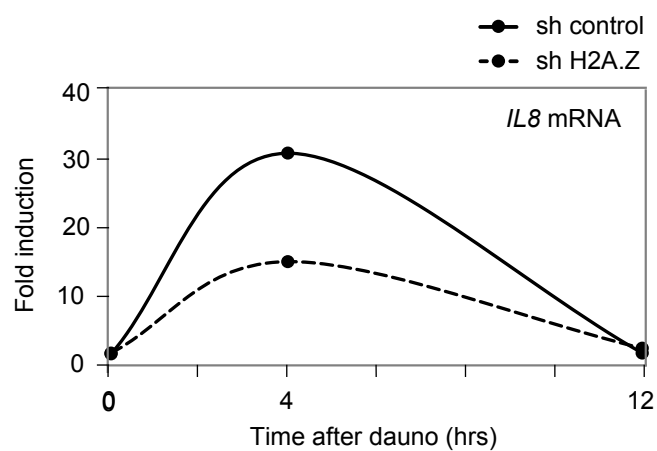

## B

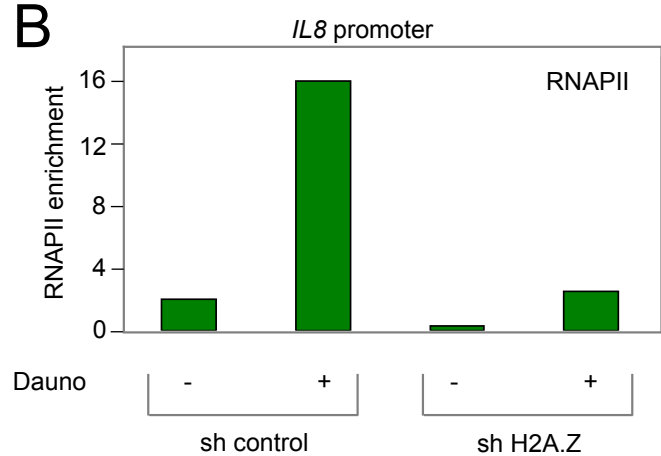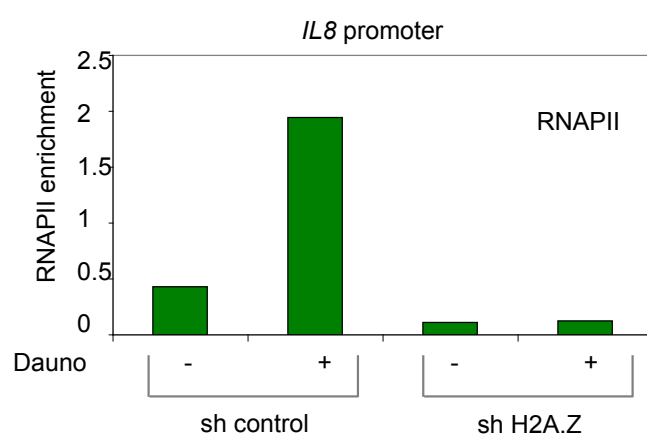

Supplement: Figure S8 — A complement to Figure 2A–2B. (A) Knockdown of H2A.Z cripples the activation of IL8 upon dauno treatment. Data from individual replicates of the experiment shown in Figure 2A. (B) Knockdown of H2A.Z cripples the recruitment of RNAPII to the IL8 promoter upon dauno treatment. Data from individual replicates of the experiment shown in Figure 2B. (0.26 MB PDF) [file pgen.1000687.s008.pdf]

Figure S9

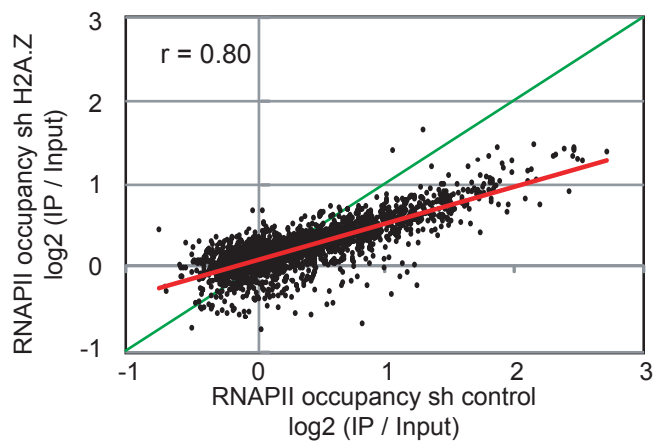

Supplement: Figure S9 — A complement to Figure 2C. Knockdown of H2A.Z causes a general decrease of RNAPII on genes as opposed to a major reshuffling of the transcriptome. RNAPII enrichment from cells treated with control shRNAs is plotted against the enrichment of RNAPII from cells treated with H2A.Z-specific shRNAs.The red line shows the trend of the data while the green line represent a slope of 1, corresponding to what would have bend expected with no global effect of H2A.Z. A massive reshuffling, on the other hand, would have generated no correlation. (0.41 MB PDF) [file pgen.1000687.s009.pdf]

Figure S10

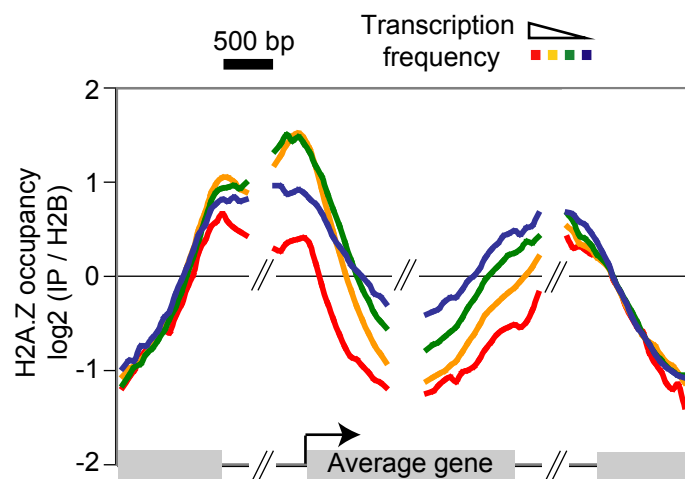

Supplement: Figure S10 — H2A.Z is depleted from transcribed open reading frames in yeast. Yeast H2A.Z ChIP-chip data from [1] were mapped on gene groups based on expression level based on [2]: The red group contains the most transcribed genes while the blue group contains the least transcribed yeast genes (662, 2964, 1345, and 1053 genes respectively). (1. Guillemette B, Bataille AR, Gevry N, Adam M, Blanchette M et al. (2005) Variant histone H2A.Z is globally localized to the promoters of inactive yeast genes and regulates nucleosome positioning. PLoS Biol 3: e384. 2. Holstege FC, Jennings EG, Wyrick JJ, Lee TI, Hengartner CJ et al. (1998) Dissecting the regulatory circuitry of a eukaryotic genome. Cell 95: 717–728.) (0.23 MB PDF) [file pgen.1000687.s010.pdf]

# Figure S11

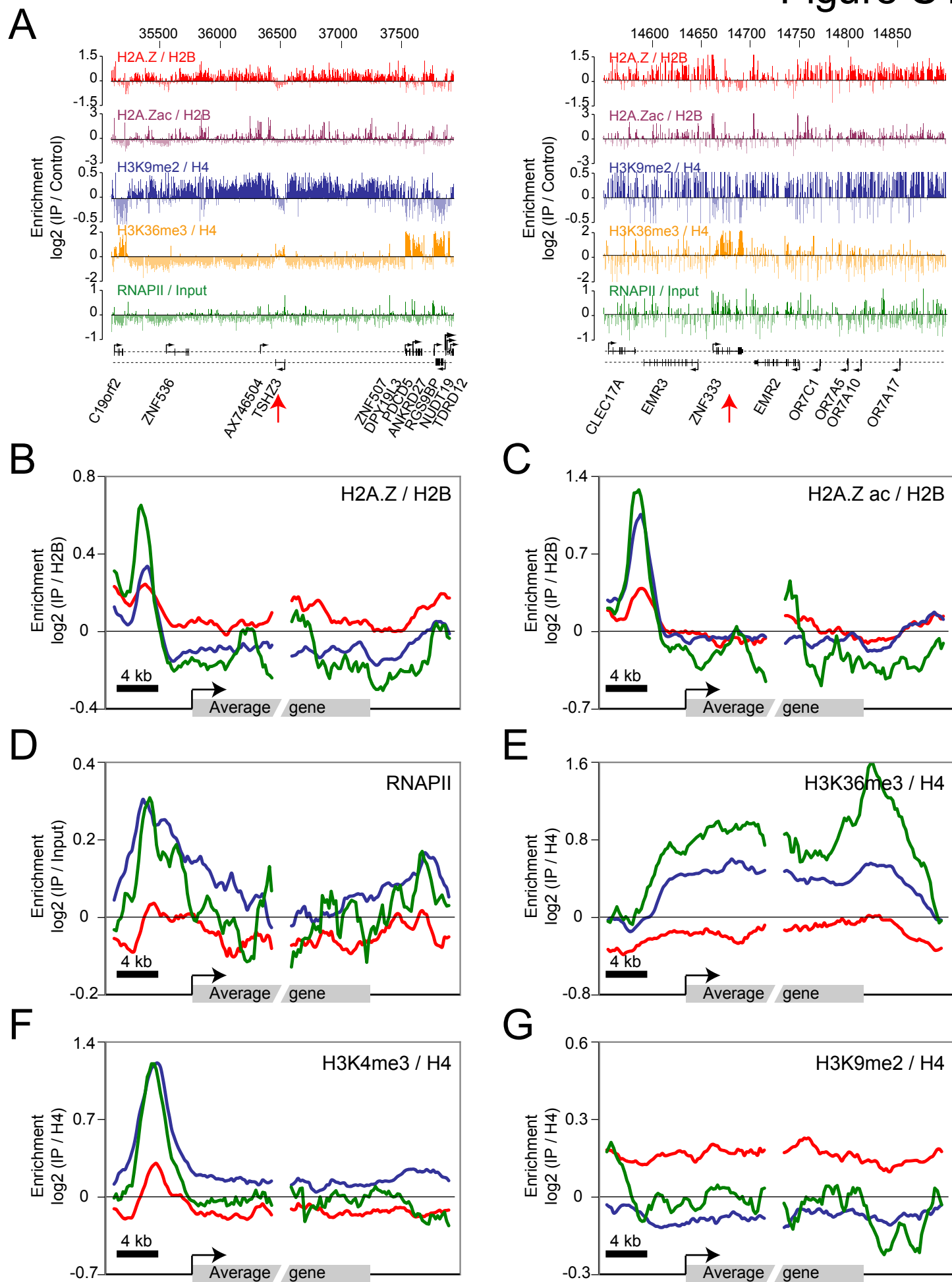

Supplement: Figure S11 — H2A.Z covers the entire length of heterochromatic genes except for genes that escape silencing. (A) Genome browser screenshots of two heterochromatic regions on chromosome 19 that contain one gene that escapes silencing (red arrows). (B–G) The average ChIP-chip signal is shown over all unique TSS inside heterochromatic regions (392 genes, red), euchromatic genes (392 genes, blue), and heterochromatic genes that escaped silencing (50 genes, green). The genes that escape silencing are defined as genes with low H3K9me2 and high H3K36me3 that are contained within a heterochromatic region. The data are shown for H2A.Z/H2B (B), acetylated H2A.Z/H2B (C), RNAPII/input (D), H3K36me3/H4 (E), H3K4me3/H4 (F), and H3K9me2/H4 (G). (1.08 MB PDF) [file pgen.1000687.s011.pdf]

Figure S12

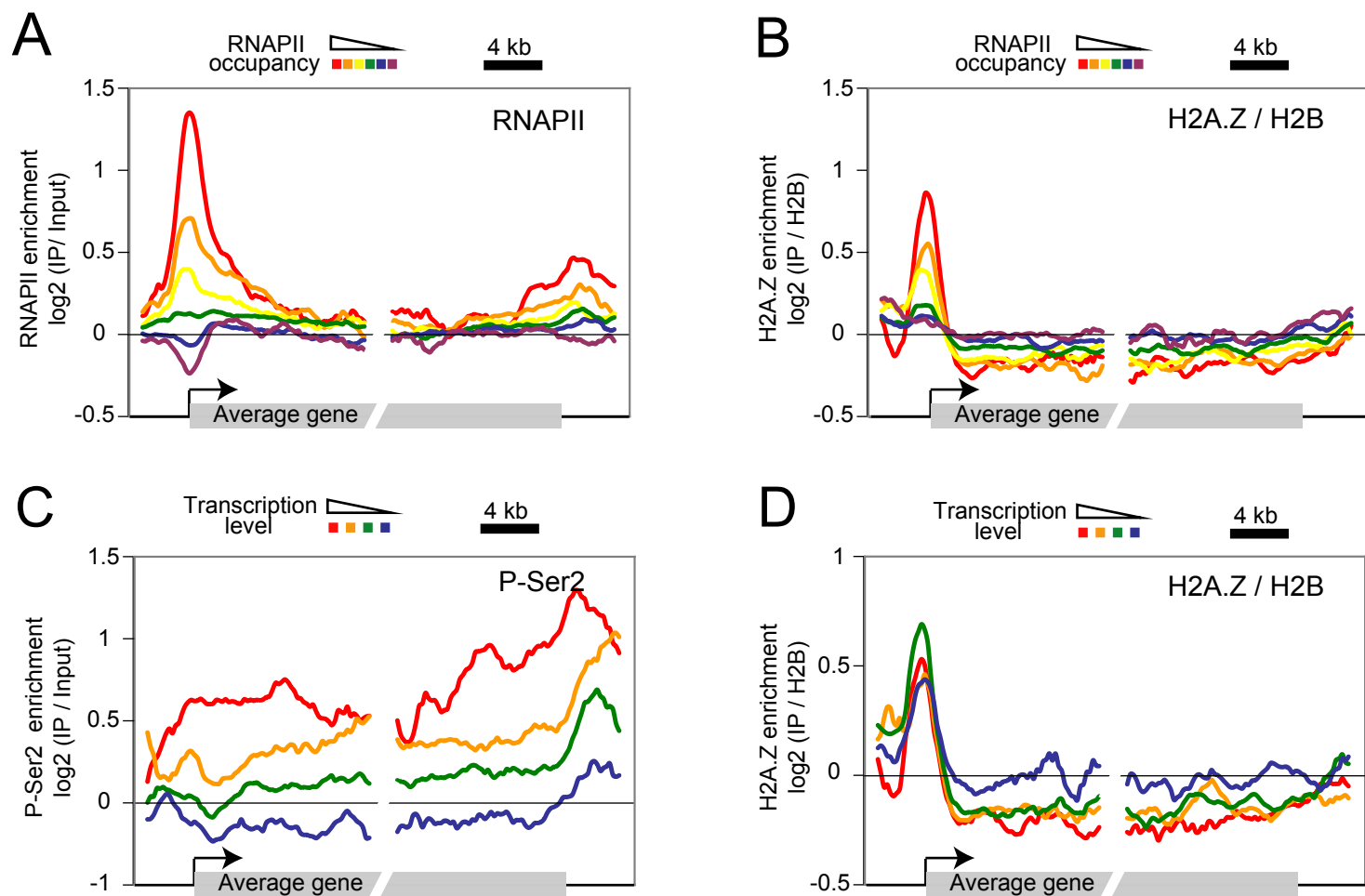

Supplement: Figure S12 — Removing the ZNF genes from our dataset does not affect the results shown in Figure 1. Because chromosome 19 contains a certain number of ZNF genes–and because these genes were shown to sometimes have a peculiar chromatin structure–we wished to repeat the analyses shown in Figure 1B–1E by first removing all ZNF genes from the dataset. Panels A, B, C, and D represent mirror analyses of panels B, C, D, and E from Figure 1 respectively, except that all ZNF genes were remove before computing the data. (0.80 MB PDF) [file pgen.1000687.s012.pdf]
