# Supplementary material for: The Euchromatic and Heterochromatic Landscapes Are Shaped by Antagonizing Effects of Transcription on H2A.Z Deposition
Source: PLoS Genet. 2009 Oct 16;5(10):e1000687. doi: 10.1371/journal.pgen.1000687 (PMC2754525; doi:10.1371/journal.pgen.1000687)
Supplement: Table S1 — Table containing the list of all experiments described in this work. (0.05 MB DOC) [file pgen.1000687.s013.doc]

**Table S1. List of all experiments described in this work.**

| **Human U2OS cells** | **Array used** | |
| --- | --- | --- |
| **Protein / Histone mark** | **chr19_244k** | **chr22_2X44k** |
| RNAPII vs input | 2 rep | 3 rep |
| P-Ser2 RNAPII vs Input | 2 rep | not done |
| H2A.Z vs H2B | 2 rep | 4 rep |
| H2A.Z vs input | not done | 2 rep |
| H2A.Z vs H4 | not done | 3 rep |
| H2A.Zac vs H2B | 2 rep | 2 rep |
| H2B vs H4 | 2 rep | not done |
| H3K36me3 vs H4 | 2 rep | 2 rep |
| H3K4me1 vs H4 | 2 rep | 2 rep |
| H3K4me2 vs H4 | not done | 2 rep |
| H3K4me3 vs H4 | 2 rep | 2 rep |
| H3K9me2 vs H4 | 2 rep | 2 rep |
| H3K9me2 vs input | not done | 2 rep |
| H4 vs input | 2 rep | not done |
| RNAP vs input with sh H2A.Z | 2 rep | not done |
| RNAP vs input with sh Ctrl | 2 rep | not done |

| ***S. cerevisiae* cells** | **Heat-shock**  **duration** | **Array used** |
| --- | --- | --- |
| **Protein / Histone mark** |  | **4 X 44k** |
| RNAPII vs input | 0 min | 2 rep |
| RNAPII vs input | 15 min | 2 rep |
| RNAPII vs input | 120 min | 2 rep |
| H2A.Z vs H2B | 0 min | 2 rep |
| H2A.Z vs H2B | 15 min | 2 rep |
| H2A.Z vs H2B | 120 min | 2 rep |
| H2A vs H2B | 0 min | 2 rep |
| H2A vs H2B | 15 min | 2 rep |
| H2A vs H2B | 120 min | 2 rep |

4 x 44k = G4493A
